# Ultra-high gradient connectomics and microstructure MRI scanner for imaging of human brain circuits across scales

**DOI:** 10.1038/s41551-025-01457-x

**Published:** 2025-07-16

**Authors:** Gabriel Ramos-Llordén, Hong-Hsi Lee, Mathias Davids, Peter Dietz, Andreas Krug, John E. Kirsch, Mirsad Mahmutovic, Alina Müller, Yixin Ma, Hansol Lee, Chiara Maffei, Anastasia Yendiki, Berkin Bilgic, Daniel J. Park, Qiyuan Tian, Bryan Clifford, Wei-Ching Lo, Stefan Stocker, Jasmine Fischer, Gudrun Ruyters, Manuela Roesler, Andreas Potthast, Thomas Benner, Elmar Rummert, Rebecca Schuster, Peter J. Basser, Thomas Witzel, Lawrence L. Wald, Bruce R. Rosen, Boris Keil, Susie Y. Huang

**Affiliations:** 1 Athinoula A. Martinos Center for Biomedical Imaging, Department of Radiology, Massachusetts General Hospital, Charlestown, MA USA; 2 Harvard Medical School, Boston, MA USA; 3 Siemens Healthineers, Erlangen, Germany; 4 Institute of Medical Physics and Radiation Protection, TH-Mittelhessen University of Applied Sciences, Giessen, Germany; 5 Center for Neurotechnology and Neurorecovery, Department of Neurology, Massachusetts General Hospital, Boston, MA USA; 6 Siemens Medical Solutions USA, Boston, MA USA; 7 Eunice Kennedy Shriver National Institute of Child Health and Human Development, National Institutes of Health, Bethesda, MD USA; 8 Q Bio Inc., San Carlos, CA USA; 9 Department of Diagnostic and Interventional Radiology, University Hospital Marburg, Philipps University of Marburg, Marburg, Germany; 10 LOEWE Research Cluster for Advanced Medical Physics in Imaging and Therapy (ADMIT), TH-Mittelhessen University of Applied Sciences, Giessen, Germany

**Keywords:** Imaging techniques, Magnetic resonance imaging

## Abstract

Defining the connectome, the complete matrix of structural connections between the nervous system nodes, is a challenge for human systems neuroscience due to the range of scales that must be bridged. Here we report the design of the Connectome 2.0 human magnetic resonance imaging (MRI) scanner to perform connectomics at the mesoscopic and microscopic scales with strong gradients for in vivo human imaging. We construct a 3-layer head-only gradient coil optimized to minimize peripheral nerve stimulation while achieving a gradient strength of 500 mT m^−1^ and a slew rate of 600 T m^−1^ s^−1^, corresponding to a 5-fold greater gradient performance than state-of-the-art research gradient systems, including the original Connectome (Connectome 1.0) scanner. We find that gains in sensitivity of up to two times were achieved by integrating a 72-channel in vivo head coil and a 64-channel ex vivo whole-brain radiofrequency coil with built-in field monitoring for data fidelity. We demonstrate mapping of fine white matter pathways and inferences of cellular and axonal size and morphology approaching the single-micron level, with at least a 30% sensitivity improvement compared with Connectome 1.0.

## Abstract (web summary)

The Connectome 2.0 MRI scanner reconstructs the fine fibres in the human brain and determines cellular and axonal size at an ultra-high resolution.

## Main

Understanding the relationship between structure and function in the human brain remains a huge challenge in human neuroscience^1^. Linking circuit structure to function is essential to uncover the principles governing human thought and behaviour. Tools are now being built to bridge the vast range of spatial scales spanned by human brain circuits: the macroscopic inter-areal connections across the whole brain at the scale of centimetres to millimetres; the mesoscopic connections between neuronal cell types on the order of hundreds of microns in size; and the microscopic connections between individual cells on the scale of nanometres to microns^2,3^. The importance of defining the structural and connectional motifs across scales in the human brain has never been more apparent, as alterations in cellular architecture and connectional anatomy are observed in animal models of neuropsychiatric disorders^4–6^ and, increasingly, in human tissue^7^. The ideal technology for probing circuit structure in the human brain would integrate across spatial scales and be sensitive to dynamic changes arising from neural plasticity, development and pathology within and across individuals.

Diffusion magnetic resonance imaging (MRI) uses the random thermal motion of water to probe the microscopic tissue environment non-invasively^8–11^. Water’s molecular diffusion profiles provide insight into microstructural features such as cell size, shape and packing density, whose cellular-scale dimensions (~µm) are orders of magnitude below the nominal voxel resolution of MRI (~mm). As a result, diffusion MRI holds great promise among non-invasive imaging techniques for probing cellular structures of any depth and location in the living human brain. The availability of higher maximum gradient strength on human MRI scanners has allowed the translation of methods previously limited to ex vivo and animal studies on small-bore systems^12–16^ to the living human brain^17–27^. However, robust mapping of tissue microstructure by diffusion MRI requires faster and stronger diffusion-encoding gradients to achieve sensitivity for probing the smallest cellular compartments.

The NIH Brain Research through Advancing Innovative Neurotechnologies (BRAIN) Initiative has invested strategically in advancing integrative neurotechnologies that push the limits of discovery to uncover how dynamic patterns of circuit structure and activity transform into cognition, emotion, perception and action in health and disease^28^. Through support from the BRAIN Initiative, we have developed the next-generation human connectomics and microstructure MRI scanner known as Connectome 2.0 (ref. ^29^) equipped with nearly double the gradient strength and triple the slew rate for human imaging. This high-performance 3 Tesla (3 T) MRI scanner was designed and optimized for studying neural tissue architecture and connectional anatomy across scales in humans. We built on the expertise that we gained during the Human Connectome Project by engineering the original 3 Tesla Connectome MRI scanner, which featured a maximum gradient amplitude of 300 mT m^−1^ (ref. ^17^) and achieved comprehensive mapping of macroscopic white matter connectional anatomy throughout the living human brain^17,18^. The original Connectome scanner provided a demonstration in living humans that strong diffusion-sensitizing gradients combined with advanced biophysical modelling enable quantification of axonal diameter and cellular-scale features with a diffusion resolution down to several microns, which is unattainable on conventional MRI scanners^19–27,30^. However, the gradient strength of the initial Connectome MRI scanner lacked sensitivity to the smallest axons that make up most white matter in the human brain^31^. Simulations and theory^27,32–34^ have shown that stronger gradients are needed to image tissue microscopic structure and map connectional anatomy at this mesoscopic scale.

These lessons served as the foundation for the development of the Connectome 2.0 scanner, an ultra-high gradient strength human MRI scanner for mesoscopic imaging of human brain microstructure and connections. Here we report on the design, construction and evaluation of each subsystem of the Connectome 2.0 scanner. We demonstrate accurate reconstruction of fine fibres deep in the brain and inference of cellular and axonal size at a microstructure-scale diffusion resolution.

## Results

### Scanner and gradient coil

**Fig. 1:**
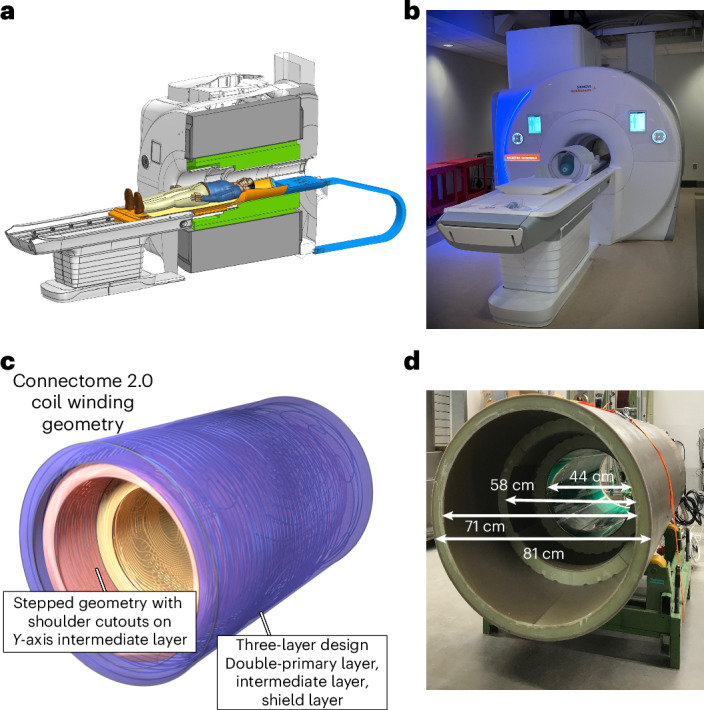
Connectome 2.0 gradient coil. **a**, Computer-aided design model of the Connectome 2.0 scanner showing the gradient coil (green), the receive and transmit coil connectors, and energy chain extending through the back of the magnet (blue) positioned on the movable table (orange). **b**, Photograph of the Connectome 2.0 scanner installed at the Massachusetts General Hospital with the 72-channel receive coil and local transmit coil positioned on the table. **c**, Schematic rendering of the gradient coil winding geometry for all coil axes. The gradient coil has a stepped geometry with shoulder cutouts on the intermediate layer. The coil consists of a double-primary layer (yellow), an intermediate layer (red) and a shield layer (blue). **d**, Photograph of the actual Connectome 2.0 gradient coil. The inner diameters of the cylinders enveloping the 3 layers are 44 cm, 58 cm and 71 cm. The outer diameter of the overall coil is 81 cm.

The Connectome 2.0 MRI scanner (MAGNETOM Connectom.X, Siemens Healthineers, Erlangen, Germany) was built as a 3 T scanner using the latest magnet technology, which provides excellent homogeneity and high stability. The choice of 3 T as the optimal field strength was informed by simulations and analyses performed for the original Connectome MRI scanner^17^, hereafter referred to as the Connectome 1.0 scanner. The essential benefits of operating at 3 T for diffusion MRI include reduced *T*_2_-relaxation-induced signal loss compared with higher field strengths (see Supplementary Fig. 1 for a detailed comparison), greater transmit field homogeneity, lower radiofrequency (RF) heat deposition, reduced vibrations and less acoustic noise. Figure 1a,b shows the designed and installed scanner.

The gradient coil was designed as an asymmetric head-only gradient to attain a target maximum gradient strength (*G*_max_) of 500 mT m^−1^ and maximum slew rate (SR_max_) of 600 T m^−1^ s^−1^ per axis, nearly doubling the maximum gradient amplitude and tripling the maximum slew rate of the original Connectome scanner (*G*_max_ = 300 mT m^−1^, SR_max_ = 200 T m^−1^ s^−1^)^17,29^ (Table 1). For reference, most clinical MRI scanners have gradient strengths between 40 and 60 mT m^−1^, with typical clinical high-performance systems achieving a *G*_max_ of 80 mT m^−1^ and SR_max_ of 200 T m^−1^ s^−1^. The performance of the Connectome 2.0 gradient system, defined as the product of *G*_max_ and SR_max_, is more than 20 times greater than that of most clinical scanners and 5 times greater than Connectome 1.0 (ref. ^29^). The Connectome 2.0 gradient coil was adapted from the 3-layer geometry of the Siemens 7 T Impulse head gradient coil (Siemens Healthineers), which targeted and achieved mesoscale functional MRI with a *G*_max_ = 200 mT m^−1^ and SR_max_ = 900 T m^−1^ s^−1^ (ref. ^35^). Figure 1c shows the coil winding geometry of the Connectome 2.0 gradient coil. To achieve the Connectome 2.0 coil’s higher gradient amplitude of 500 mT m^−1^ compared with the Impulse coil’s 200 mT m^−1^, the current density was increased by doubling the primary layer windings for all three axes, as opposed to only along the *Z* axis for the Impulse gradient. This modification required additional fine-tuning of the wire geometry to compensate for reduced efficiency. Connectome 2.0 gradient’s lower target slew rate of 600 T m^−1^ s^−1^ compared with the Impulse gradient’s 900 T m^−1^ s^−1^ provided a path to raise the gradient strength to 500 mT m^−1^, essentially by trading current density (leading to higher mT m^−1^) for wire length (leading to higher inductance and lower slew rate). While both a linear magnetic field gradient in the field-of-view (FOV) and an efficient shielding towards the cryostat could be produced by the primary and secondary layers alone (that is, the standard layers of a gradient coil), the additional intermediate layer of the Connectome 2.0 coil gradient facilitated additional degrees of freedom to optimize torque and force balancing without compromising the gradient field performance^35^.

**Table 1:** Connectome 2.0 gradient coil performance

	Connectome 2.0	Connectome 1.0
Patient bore at isocentre (cm)	40	56
Gradient coil inner diameter (cm)	44	61
Gradient coil outer diameter (cm)	81	89
Length (cm)	160	154
Gradient performance along each axis		
Maximum amplitude (mT m^−1^)	500	300
Maximum slew rate (T m^−1^ s^−1^)	600	200
Gradient power amplifiers		
Number of GPAs per axis	2	4
Peak current (A)	1,200	900
Peak voltage (V)	2,250	2,250
Gradient coil sensitivity, *η* (mT m^−1^ A^−1^)	0.42	0.34
Inductance, *L* (μH) (*x*, *y*, *z*)	2,250, 2,450, 1,800	4,800, 5,600, 5,350
Direct current resistance per axis (Ω)	0.28	0.42
Linearity over 20-cm sphere (%)	*X*: 6.7, *Y*: 8.2, *Z*: 11.7	*X*: 6.0, *Y*: 6.3, *Z*: 5.2
Coil mass (kg)	1,125	~1,400
Net force (kN)	0.13	0.21
Net torque (Nm)	75	0
*G*_r.m.s._ at 100% direct current (mT m^−1^)		
Single axis (mT m^−1^)	175	N/A
All 3 axes (mT m^−1^)	150	N/A
Post-compensated eddy current (%)	0.075	0.040
Acoustics: NEMA max. noise (dB(A))	116	114
Active shims	1st and 2nd order	1st and 2nd order

Performance specifications of the Connectome 2.0 head gradient coil compared with the Connectome 1.0 gradient coil. Linearity value (%) is defined as the worst-case percentage deviation of the achieved *B*_*z*_ field from the ideal *B*_*z*_ field, *B*_z-ideal_, at any point over a 20-cm sphere, that is, $100\max \{\left|{B}_{{\rm{z}}}-{B}_{{\rm{z}}-{\rm{ideal}}}\right|\}/\max \{\left|{B}_{{\rm{z}}-{\rm{ideal}}}\right|\}$^36^. *G*_r.m.s._ denotes the root mean square of the gradient waveform.

**Fig. 2:**
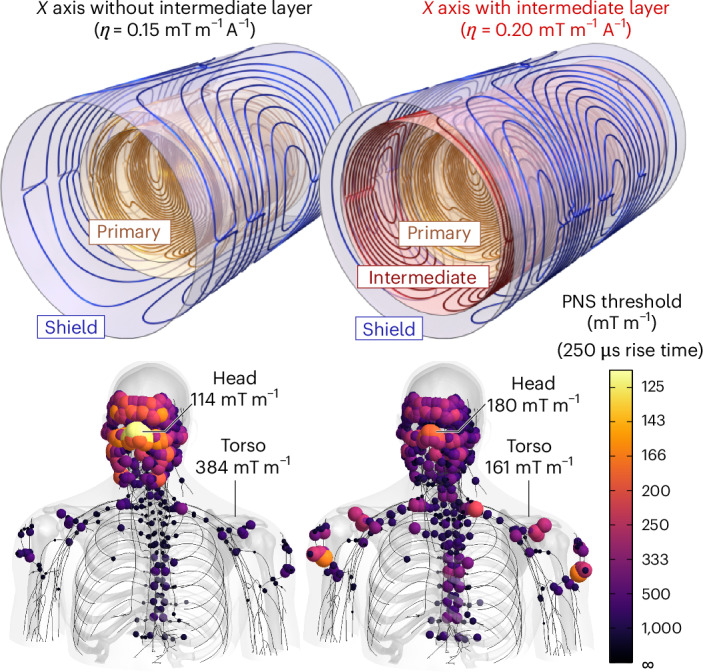
Illustration of two *X*-axis coil winding designs without and with the intermediate coil winding layer (red) and the resulting local PNS threshold maps. Every coloured sphere corresponds to a stimulation site, with colour and size corresponding to the local PNS threshold (yellow = low threshold, that is, easy to stimulate). The 2-layer design (double-primary layer, shield layer) on the left led to predominant stimulation of facial nerves, resulting in overall low PNS thresholds (114 mT m^−1^) and thus low coil usability. The inclusion of an intermediate winding layer (right) allowed for balancing of PNS between the face and torso, thus raising the worst-case thresholds by 40%.

The Connectome 2.0 and Impulse head gradient coils both incorporated peripheral nerve stimulation (PNS) modelling in the design phase to raise the PNS thresholds, recognizing that PNS would impose biological limits to attaining usage of the full *G*_max_ and SR_max_ of these high-performance systems. The additional intermediate layer opened degrees of freedom to achieve PNS balancing and raise nerve stimulation thresholds to maximize the usable gradient performance space^36^. The concept of PNS balancing is shown in Fig. 2. The conventional 2-layer gradient coil geometry (double-primary layer, yellow, plus shield layer, blue) resulted in low PNS thresholds (114 mT m^−1^) in the facial area and high thresholds in the torso (384 mT m^−1^). The inclusion of an intermediate coil winding layer (red) allowed for the reshaping of the coil’s magnetic fields and better PNS balancing. This design led to raised PNS thresholds in the facial area (180 mT m^−1^) and reduced PNS thresholds in the torso (161 mT m^−1^), thus raising the overall worst-case PNS thresholds by 41% (114 mT m^−1^ to 161 mT m^−1^). The added layer also enabled the target gradient coil sensitivity (gradient strength per unit current) of 0.42 mT m^−1^ A^−1^ in the final coil design (Fig. 1c).

The Connectome 2.0 gradient coil followed a stepped design with shoulder cutouts in the *Y* axis’ intermediate layer to accommodate a range of human participant sizes. The inner diameters of the cylinders enveloping the 3 layers are 44 cm for the inner layer, 58 cm for the middle layer and 71 cm for the outer layer (Fig. 1d). The diameters of the patient bore liner are 40 cm for the inner layer, 56 cm for the middle layer and 60 cm for the outer layer. The smaller inner diameter of the Connectome 2.0 head gradient coil (44 cm) enabled higher gradient coil sensitivity compared with the gradient coil of the Connectome 1.0 scanner (gradient coil inner diameter of 61 cm) (Table 1). The sensitivity of the Connectome 2.0 head gradient coil is about 4 times higher than that of whole-body gradient coils and 30% higher than those of other high-performance MRI scanners designed for brain microstructure imaging^37^. The higher gradient sensitivity reduces the required number of gradient amplifiers. Specifically, the Connectome 2.0 gradient system is driven by two gradient power amplifiers (GPAs) per axis (Table 1), in contrast to four GPAs per axis in the Connectome 1.0 scanner. The wiring pattern of each gradient coil axis is split into two partial coils comprising two half cylinders, such that the *X*, *Y* and *Z* partial coils have as little mutual inductive coupling as possible. Each partial coil consists of a three-layer set-up, and each partial coil is connected to a single GPA. Ultimately, each GPA drives a partial coil that encompasses half of the volume contained inside the gradient coil. Each GPA in Connectome 2.0 is capable of a peak current of 1,200 A and peak voltage of 2,250 V, producing 33% more power than the gradient power amplifiers of the Connectome 1.0 scanner. The gradient coil utilizes a novel direct cooling technique using stainless-steel tubing surrounded by conductive copper filaments, allowing the coil to be operated at high-duty cycles with minimal heating. Applying 100% direct current for a single axis at a time, we found that the Connectome 2.0 gradient coil could safely sustain a maximum gradient strength of 175 mT m^−1^, reaching the upper temperature bound of 85 °C at steady state, while remaining within safe operating limits of the gradient coil. Similarly, when applying 100% direct current for all 3 gradient axes simultaneously, the maximum gradient strength was 150 mT m^−1^ to attain a steady state temperature of 85 °C. The maximum power dissipation was 70 kW for a single axis and 190 kW for all 3 axes combined. Maximum technical sound levels, following the MGAN procedure of NEMA MS4 regulation, reached a value of 116 dB(A). As conventional clinical MRI scanners typically reach values between 110 and 125 dB(A), no specific acoustic measures need to be considered for the Connectome 2.0 scanner. The use of conventional earplugs with a minimum attenuation of 20 dB is sufficient to stay within the safety limits.

**Fig. 3:**
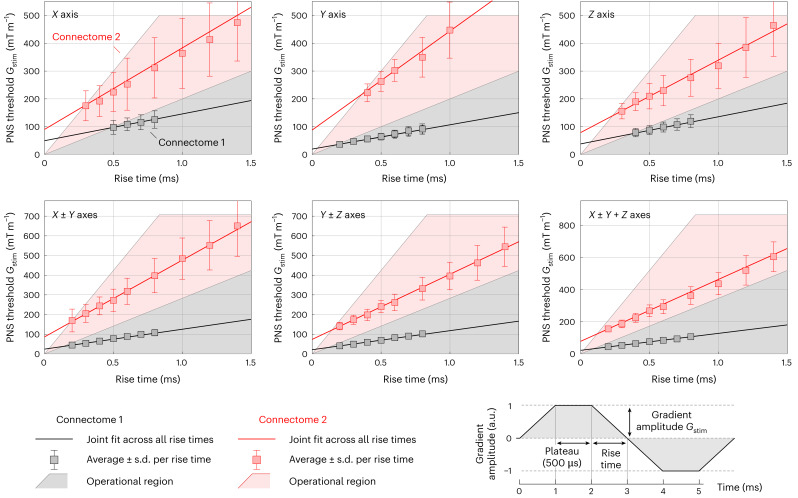
Experimental PNS threshold curves for the Connectome 2.0 head-only gradient coil (red) and the original Connectome coil (grey). The raised threshold curves of the Connectome 2.0 coil, together with the increased hardware performance space determined by *G*_max_ and SR_max_ (red-shaded area), increase the performance space that can be safely used in humans compared with the Connectome 1.0 gradient coil. The number of human participants in this volunteer study was *n* = 29. Note that the thresholds shown here are population mean PNS thresholds, not those from the SAFE model. We use *G*_stim_ to reflect the smallest stimulation gradient amplitude (zero-to-peak convention).

The worst-case linearity errors over a 20-cm-diameter spherical volume for the Connectome 2.0 gradient coil are 6.7%, 8.3% and 11.7% for the *X*, *Y* and *Z* axes, respectively. For the same axes, the errors for the Connectome 1.0 coil are 6.0%, 6.3% and 5.2%, while for the Impulse gradient coil, the errors are 6.9%, 7.0% and 9.0% (ref. ^35^). The greater nonlinearity (smaller linearity region size) of the Connectome 2.0 gradient coil was a necessary trade-off to achieve the targeted higher maximum gradient strength compared with both the Impulse and Connectome 1.0 gradient coils. Ultimately, an 11.7% nonlinearity degree along the *Z* axis does not have a substantial effect on the image quality, as the geometric distortion can be effectively corrected using the spherical harmonic expansion coefficients provided by the vendor (see Supplementary Fig. 2). However, there is an unrecoverable loss of spatial resolution at the edge of the imaging region due to uniformity (pixel size) errors, which are not corrected by the distortion correction algorithm. The Connectome 2.0 gradient coil was characterized by the gradient impulse response function (GIRF) method^38^. A combination of blips and sweep pulses^39^ were used for each axis for GIRF characterization. The magnetic field responses were measured using a 16-channel dynamic field camera (Skope, Zurich, Switzerland)^40^. GIRF responses for all three axes and the measured cross-responses (up to a second order)^36^ are shown in Supplementary Figs. 3 and 4, respectively. The PNS thresholds of the constructed Connectome 2.0 gradient coil were measured in 29 healthy volunteers with written informed consent. The stimulation study used a trapezoidal pulse train with 128 cycles, varying rise times (50–3,000 µs) and constant flat-top duration (500 µs). Figure 3 summarizes the population average PNS thresholds for single-axis (*X*, *Y*, *Z*) and combined-axes (*X* ± *Y*, *Y* ± *Z*, *X* ± *Y* + *Z*) modes (see Supplementary Table 1 for additional details). PNS thresholds are reported as the smallest stimulation gradient amplitude as a function of the trapezoidal rise time. For comparison, we also show the threshold curves of the Connectome 1.0 gradient coil. The Connectome 2.0 head-only coil achieved between 2.4 to 4.2 times greater PNS thresholds than the Connectome 1.0 gradient coil and 2.5 to 5.4 times greater than the PNS thresholds of state-of-the-art clinical MRI scanners. PNS thresholds are also compared to those from a high-performance MRI scanner, GE MAGNUS^37^ (Supplementary Fig. 5). Practically, the Connectome 2.0 scanner can routinely achieve maximum gradient strengths of 500 mT m^−1^ and slew rates of 600 T m^−1^ s^−1^ for the diffusion-encoding gradients, limited only by the PNS thresholds described here.

### Radiofrequency coils

**Fig. 4:**
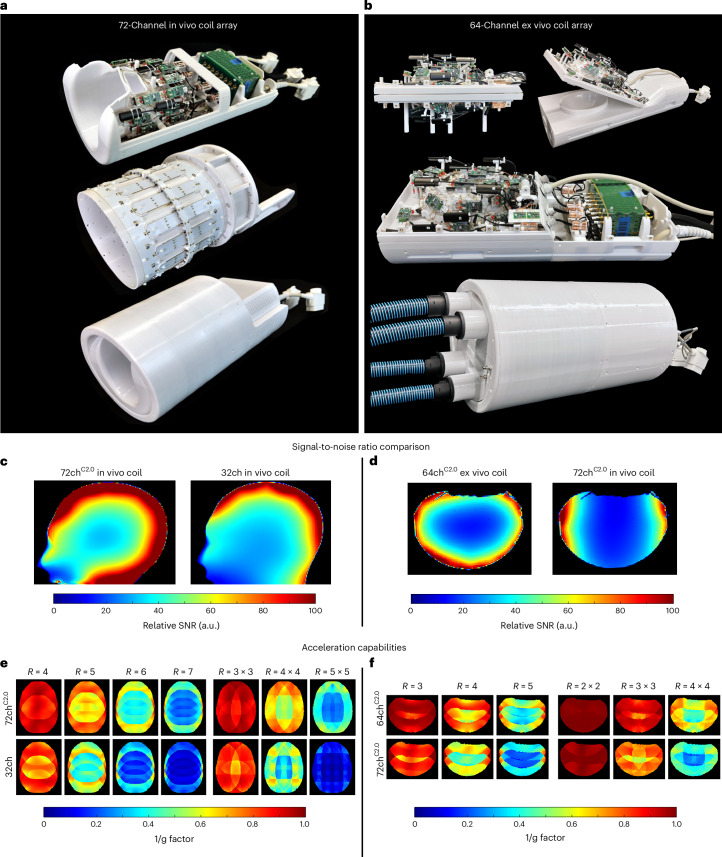
Radiofrequency coils tailored for high-fidelity in vivo and ex vivo mesoscale brain mapping. **a**, Photographs of the 72-channel in vivo receive array (top), birdcage transmit coil (middle) and integrated coil assembly (bottom). Field probes (16-channel ^19^F) are accurately positioned throughout the receive array to capture higher-order field terms. **b**, Photographs of the 64-channel ex vivo whole-brain coil (top and middle) and integrated coil assembly with ventilation tubes for temperature control (bottom). **c**, SNR maps of the 72-channel (72ch) in vivo coil compared to a 32-channel (32ch) standard coil. **d**, SNR maps of the 64-channel (64ch) ex vivo coil compared to the 72-channel in vivo coil. **e**, Image-encoding capability for accelerated imaging represented as noise amplification in g-factor maps (1/g) of the 72-channel in vivo coil compared to a 32-channel standard coil at different levels of acceleration *R*. **f**, g-factor maps of the 64-channel ex vivo coil compared to the 72-channel in vivo coil. C2.0, Connectome 2.0.

Pushing the sensitivity limits for ultra-high diffusion-weighting and high-spatial resolution mesoscale acquisitions on the Connectome 2.0 scanner required improved MRI signal reception capabilities^41–43^. Strong diffusion-encoding gradient pulses generate higher-order eddy current fields in the magnet cryostat that require compensation by concurrent field monitoring^40,44,45^. To achieve both goals, a 72-channel in vivo receive (Rx) head coil and a 64-channel ex vivo Rx brain coil array were designed and constructed (Fig. 4a,b). The 72-channel head coil offers full coverage of the cerebral hemispheres, cerebellum and brainstem. The 64-channel ex vivo array coil accommodates whole brain specimens to be imaged at submillimetre spatial resolution for multiscale validation studies of connectional anatomy and tissue microstructure in the human brain. The ex vivo coil incorporated an embedded temperature probe system that monitors a forced air-cooling circulation system to ensure the accuracy and reproducibility of long diffusion MRI measurements independent of the scanner’s environmental temperature. The space constraints of the Connectome 2.0 scanner’s gradient coil and concerns regarding heating and transmit efficiency necessitated the design of a local transmit coil for each respective receive coil, with both the gradient coil and the transmit coil utilizing their own RF shielding. Thus, each Rx coil array was equipped with its own dedicated local transmit (Tx) coil, such that the entire coil assembly could be placed in situ on the patient table’s head end. The 72-channel in vivo coil results in a 1.5-fold improvement in signal-to-noise ratio (SNR) in the peripheral regions of the phantom (corresponding to the cortical regions of the brain) and 5% in the central region when compared with a standard 32-channel head coil, the most used head coil on the Connectome 1.0 scanner^27,46–48^ (Fig. 4c). The constructed 72-channel in vivo head coil provides lower noise amplification factors during undersampled accelerated image acquisitions (Fig. 4e), providing at least an additional unit of acceleration for a given noise amplification factor compared with the 32-channel coil. The constructed 72-channel in vivo coil also provides superior SNR and acceleration capabilities compared with a 64-channel in vivo coil built for the Massachusetts General Hospital (MGH) Connectome 1.0 scanner (Supplementary Fig. 6). The 64-channel ex vivo coil was compared directly to the Connectome 2.0’s 72-channel in vivo coil. It outperforms the larger 72-channel in vivo coil by a factor of 1.73 when the average SNR is measured over representative slices along the 3 anatomical axes (Fig. 4d). The greatest improvement in SNR is found in the periphery of the phantom, especially in regions where the 64-channel’s array detector structure is placed closer to the sample.

Each Rx array was outfitted with a 16-channel dynamic field monitoring system (Skope, Zurich, Switzerland) (Supplementary Fig. 7a). This allowed measurement of the deviations of the imaging *k*-space trajectory of the diffusion-weighted images (DWIs) as a function of the diffusion weighting and direction in real time (Supplementary Fig. 7b). These deviations occurred due to long-time constant eddy currents from the preceding diffusion-encoding gradients^49^. Higher-order field terms were also substantial and diffusion-direction encoding dependent (Supplementary Fig. 7c). Phase differences between consecutive echo-planar imaging (EPI) echoes, a major source of Nyquist ghosting, exhibited nonlinear spatial patterns and were dependent on the diffusion-encoding direction (Supplementary Fig. 8a). Standard methods for Nyquist ghosting correction methods used on conventional MRI scanners assume one-dimensional linear models for phase modulations^50^ and lead to prominent ghosting in the reconstructed images (Supplementary Fig. 8b). Advanced nonlinear ghosting removal techniques such as the dual-polarity GeneRalized Autocalibrating Partial Parallel Acquisition (GRAPPA) method^51^ and, to a greater extent, concurrent field monitoring-based image reconstruction^44^, were effective in mitigating the Nyquist ghosting.

### Improvements in SNR for diffusion MRI

**Fig. 5:**
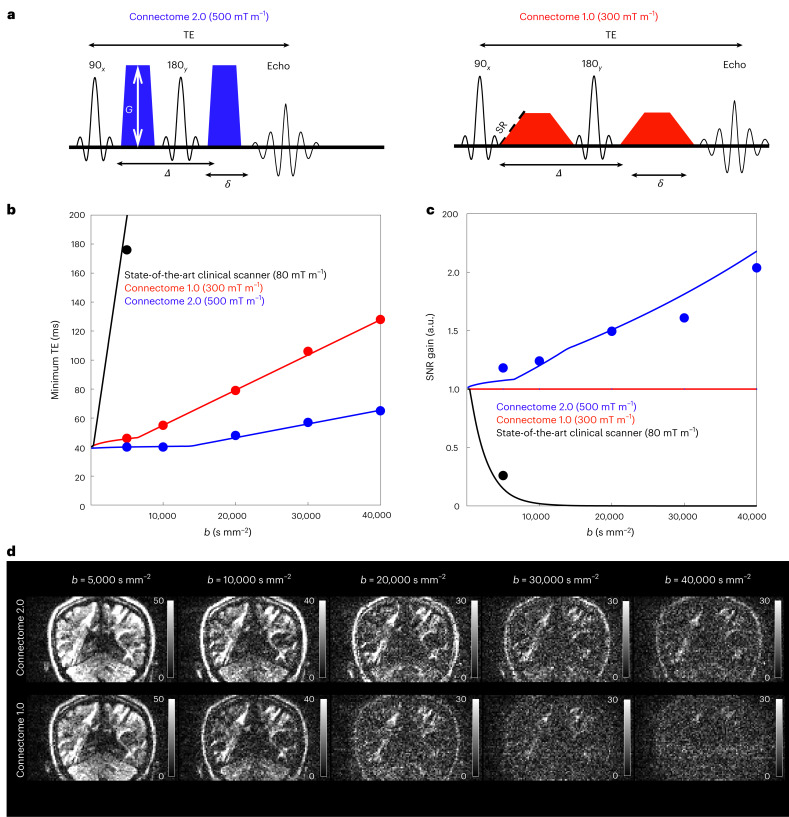
Comparison of SNR performance of representative protocols on the Connectome 2.0 scanner. **a**, Compared with the Connectome 1.0 protocol (red), the Connectome 2.0 protocol (blue) uses the higher maximal gradient strength (*G*_max_ = 500 mT m^−1^) and slew rate (SR_max_ = 600 T m^−1^ s^−1^) to shorten the diffusion time *Δ* and diffusion gradient pulse width *δ*, leading to shorter echo times TE and higher SNR for any given diffusion weighting *b*-value. **b**, Compared with the Connectome 1.0 protocol (red), the minimal TE required to achieve a given *b*-value is shorter when using the Connectome 2.0 protocol (blue). The data points are the actual sequence parameters and the curves are the predictions based on a sequence simulator. **c**, The TE shortening enabled by the Connectome 2.0 gradients yields an SNR gain of ~1.2–2 with respect to the Connectome 1.0 protocol at the highest *b*-values. The data points are the SNR measurements averaged in the cerebral white matter. The curves are the theoretical prediction using a mono-exponential decay (assuming *T*_2_ = 80 ms in white matter). **d**, DWIs demonstrate the visible SNR gains provided by the Connectome 2.0 protocol over the Connectome 1.0 protocol for *b*-values from 5,000 to 40,000 s mm^−2^.

The Connectome 2.0 scanner’s higher gradient strength and slew rate offer a substantial boost in SNR for diffusion MRI over the Connectome 1.0 and state-of-the-art clinical scanners. They enable reductions in diffusion time *Δ* and pulse width *δ* for the same diffusion-encoding *b*-value (Fig. 5a–c). Consequently, the echo time (TE) is shortened, reducing *T*_2_-relaxation-induced signal loss and improving SNR over a wide range of *b*-values (Fig. 5d). Sequence simulations for the Connectome 2.0 scanner predicted the minimum achievable TE with respect to *b*-value for different maximum gradient strength and slew rate configurations (Fig. 5b) corresponding to a state-of-the-art clinical scanner protocol (*G*_max_ = 80 mT m^−1^, SR_max_ = 200 T m^−1^ s^−1^), Connectome 1.0 protocol (*G*_max_ = 300 mT m^−1^, SR_max_ = 200 T m^−1^ s^−1^) and Connectome 2.0 protocol (*G*_max_ = 500 mT m^−1^, SR_max_ = 600 T m^−1^ s^−1^). In vivo measurements on the Connectome 2.0 scanner showed that the actual TE (Fig. 5b) and SNR (Fig. 5c) were in good agreement with the predicted values. Compared with the Connectome 1.0 protocol, reductions in TE ranging from 13% to 50% were achievable using the Connectome 2.0 protocol. Compared with the clinical protocol, TE reductions were at least 77% for the lowest *b*-value evaluated, *b* = 5,000 s mm^−2^, which is a considerably strong diffusion weighting to achieve on such a system. These reductions in TE translated into SNR gains of up to 2-fold compared with Connectome 1.0 at the highest *b-*value evaluated*, b* = 40,000 s mm^−2^, and ranging from 4-fold at *b* = 5,000 s mm^−2^ to more than an order of magnitude compared with the clinical protocol, which could only be predicted theoretically at *b*-values higher than 5,000 s mm^−2^ due to excessive experimental SNR loss.

### In vivo human brain high-spatial-resolution tractography

**Fig. 6:**
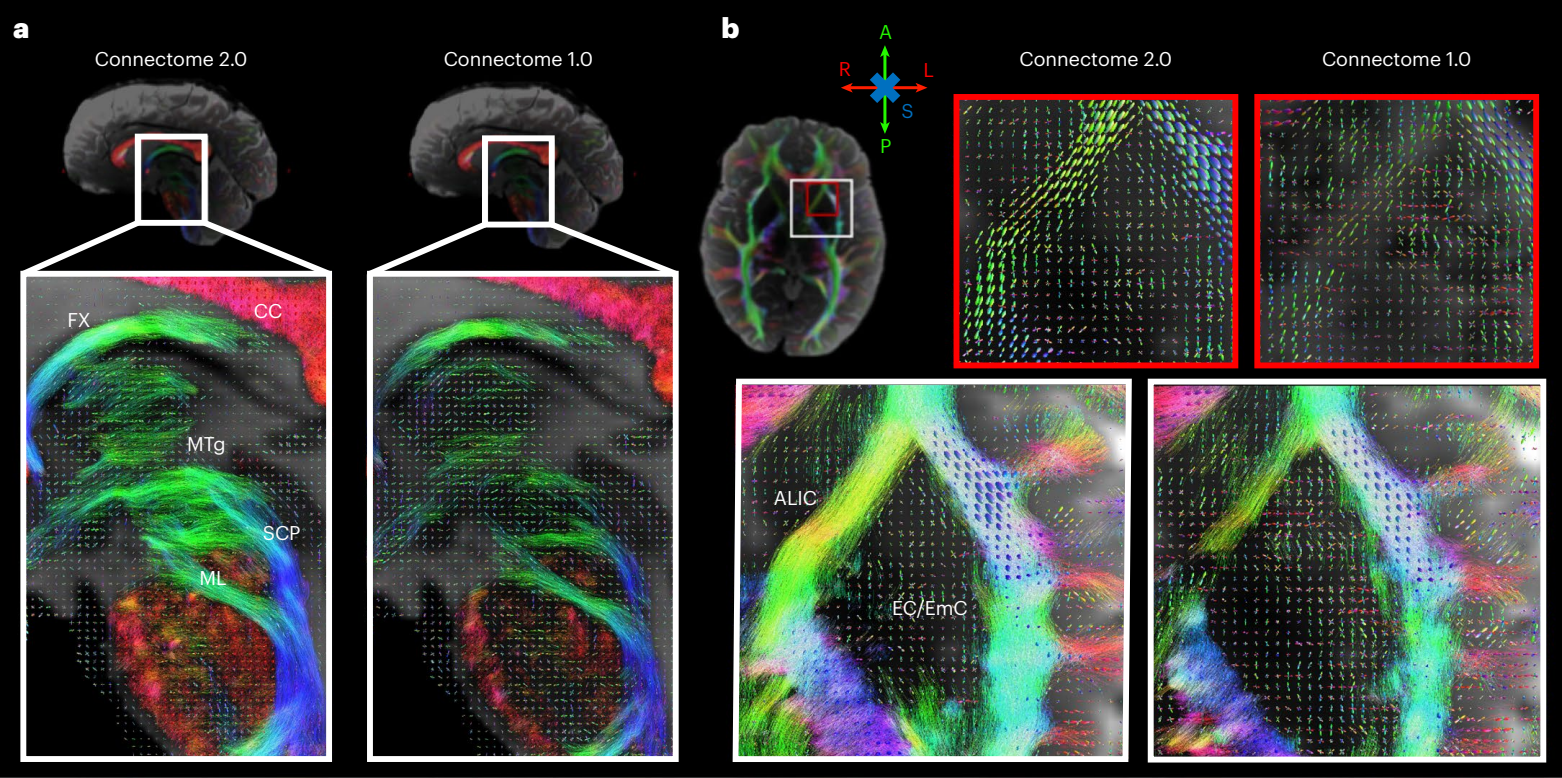
High-resolution diffusion tractography and fibre orientations on Connectome 2.0. **a**, Closeups of the midline sagittal view for Connectome 2.0 (left) and Connectome 1.0 (right) protocols, showing diencephalic and brainstem pathways. Tractography results are shown superimposed onto the underlying fibre orientation distribution functions (fODFs). **b**, Closeups of the tractography reconstruction of the inferior internal capsule and claustrum for both protocols (bottom row). fODFs are shown for a smaller region on the anterior limb of the internal capsule indicated by the red square. CC, corpus callosum; FX, fornix; ML, medial lemniscus; MTg, mammillo-tegmental tract; SCP, superior cerebellar peduncle; ALIC, anterior limb of internal capsule; EC, external capsule; EmC, extreme capsule. L/R, left/right; A/P, anterior/posterior; I/S, inferior/superior.

Due to limitations in SNR, in vivo DWIs are typically acquired at low spatial resolution with voxel sizes of 1.5–2 mm isotropic^17^, making it impossible to resolve the finest fibre bundles. For example, clinically relevant^52,53^ deep brain fibre pathways representing therapeutic targets in psychiatric and motor disorders^54^ are smaller than 2 mm in diameter^55^. We previously showed that visualizing these fibres required very high-spatial-resolution DWIs (760 μm isotropic) acquired across nine 2-h sessions on Connectome 1.0 (refs. ^56,57^). Such an acquisition would be impractical in individual patients. To demonstrate the benefits of improved SNR on Connectome 2.0 for resolving these small fibre tracts in an individual participant, we acquired DWIs at 1 mm isotropic spatial resolution within 30 min at *b* = 1,000 and 2,500 s mm^−2^ in a healthy volunteer using Connectome 2.0 and 1.0 protocols on the Connectome 2.0 scanner. As a result of the 50% SNR gain due to TE shortening at high *b*-value, diffusion tractography could delineate fine diencephalic fibre tracts, such as the mammillo-tegmental tract, only on the Connectome 2.0 dataset (Fig. 6a), and with more robust reconstruction of the internal, external and extreme capsules (Fig. 6b) compared with the Connectome 1.0 protocol.

### In vivo human brain tissue microstructure

**Fig. 7:**
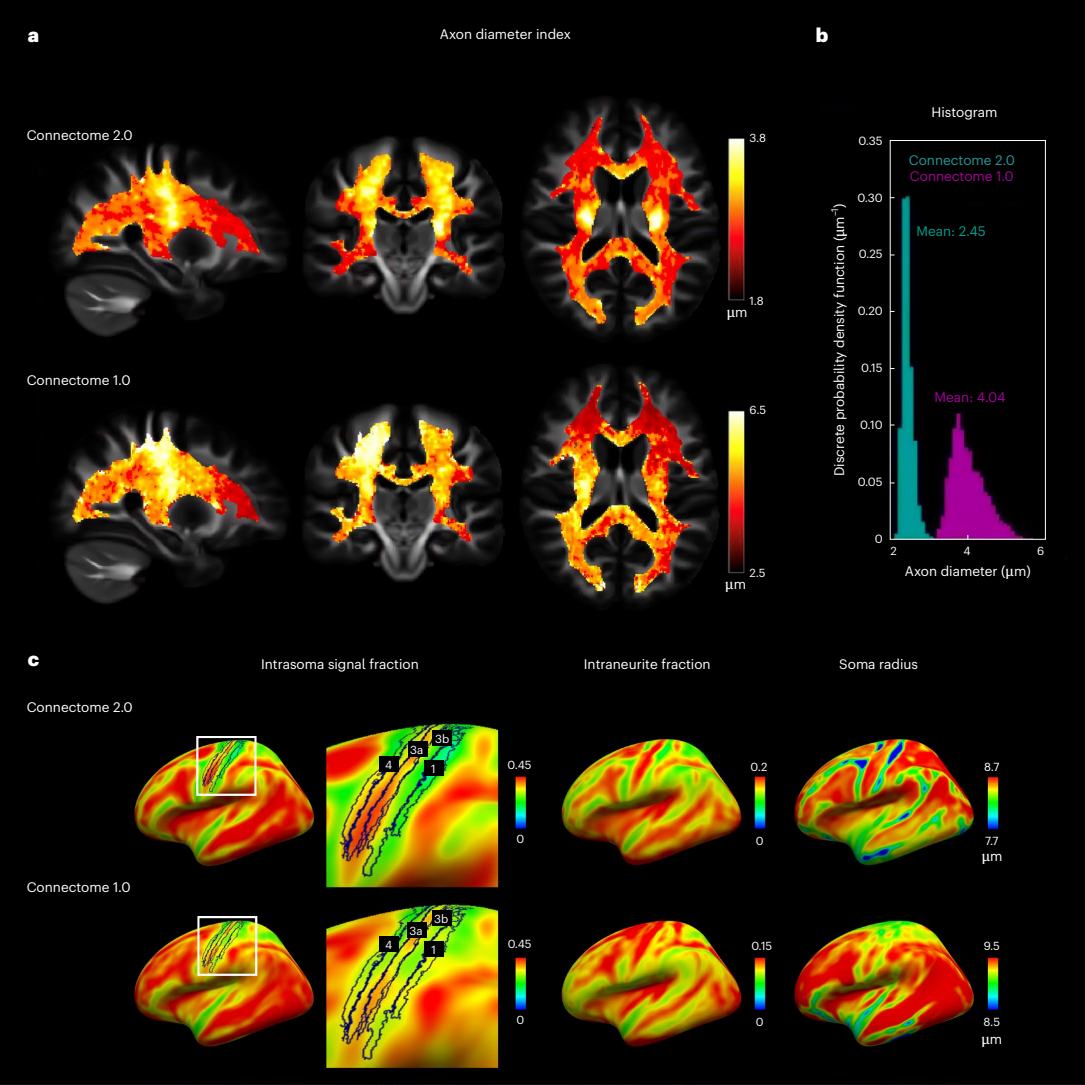
In vivo human brain tissue microstructure results. White and grey matter microstructural estimates obtained from biophysical modelling of diffusion MRI data acquired in 10 age- and sex-matched healthy adults scanned on Connectome 2.0 and Connectome 1.0 scanners. **a**, Axon diameter index maps estimated from the AxCaliber-SMT model averaged across individuals. **b**, Histogram of axon diameter indices from Connectome 2.0 and Connectome 1.0 extracted from a representative region of interest in the posterior corona radiata. **c**, Estimates of intrasoma signal fraction (left), intraneurite signal fraction (centre) and soma radius (right) from fitting of the SANDI model. SANDI estimates were averaged across individuals and projected onto the cortical surface. Inset: intrasoma signal fraction demonstrates estimates within sensorimotor cortex, delineated as Brodmann areas 4, 3a, 3b and 1.

The diffusion length in biological tissues is commensurate with axonal and cellular size at the diffusion times accessible by MRI^8,58^, which enables the evaluation of salient features of brain tissue microgeometries^59^, such as axonal diameter^14,16,21,27,60^ and cell body (soma) radius^30^, through biophysical modelling of the diffusion MRI signal. Axonal diameter is a key determinant of conduction velocity^61–64^, and variations in cell body size are related to neurodegeneration and aging^65^. Connectome 2.0 accesses shorter diffusion length scales that capture a greater proportion of small-diameter axons, previously only measurable on small-bore MRI scanners^27,66,67^. The resolution limit is roughly proportional^33^ to *G*_max_^−1/2^, suggesting an improvement in the smallest detectable axonal diameter by at least 30% using *G*_max_ = 500 mT m^−1^ compared with *G*_max_ = 300 mT m^−1^. Figure 7 shows that Connectome 2.0 achieves improvements in diffusion resolution for assessing axonal (Fig. 7a,b) and cellular size (Fig. 7c) by up to 40% compared with Connectome 1.0, leveraging the increased sensitivity afforded by the better gradients and receive coils. For example, fitting the Axonal Caliber-Spherical Mean Technique (AxCaliber-SMT) model^21^ to diffusion MRI data acquired on Connectome 2.0 using *G*_max_ = 500 mT m^−1^ and Connectome 1.0 using *G*_max_ = 300 mT m^−1^, the estimated axonal diameters were 2.45 ± 0.15 and 4.04 ± 0.48 μm in the posterior corona radiata, respectively (Fig. 7b). Thus, the Connectome 2.0 scanner provides both a systematically lower diameter measure, indicating sensitivity to smaller axons that were not captured by the Connectome 1.0, as well as lower standard deviation estimate. The higher SNR afforded by Connectome 2.0 provides greater precision of axonal diameter estimates, which are more consistent between the two hemispheres compared with Connectome 1.0 (Supplementary Fig. 9).

Similarly, fitting the Soma and Neurite Density Imaging (SANDI) model^30^, greater contrast was observed in the estimation of intrasoma signal fraction between cortical regions on Connectome 2.0 than on Connectome 1.0. For example, on Connectome 2.0, Brodmann area 3a showed the highest intrasoma signal fraction (0.36 ± 0.02) relative to other sensorimotor cortex regions (Brodmann areas 4, 3b and 1) (false discovery rate (FDR)-corrected *P* < 0.001). Primary motor cortex (Brodmann area 4) (0.32 ± 0.02) displayed remarkably higher intrasoma signal fraction than Brodmann area 3b (0.27 ± 0.03) and Brodmann area 1 (0.21 ± 0.02) (FDR-corrected *P* < 0.001). Similarly, the intrasoma signal fraction in Brodmann area 3b was higher than that in Brodmann area 1 (FDR-corrected *P* = 0.001). These findings were consistent with observations from previous histological studies^68^ (Supplementary Fig. 10), and less pronounced on Connectome 1.0 (Fig. 7c), which only exhibits a higher intrasoma signal fraction in Brodmann area 3a (0.33 ± 0.07) relative to other regions (FDR-corrected *P* < 0.004) and comparable intrasoma signal fraction to the other areas. Connectome 2.0 also showed sensitivity to a greater proportion of smaller cells, with estimated soma radii on Connectome 2.0 and Connectome 1.0 being 8.52 and 9.26 μm in overall cortical grey matter, respectively (*P* < 0.001).

## Discussion

The centrepiece of the Connectome 2.0 scanner technology is the head gradient coil, which delivers a *G*_max_ of 500 mT m^−1^. These strong gradients and fast slew rates on Connectome 2.0 improve sensitivity for high-spatial resolution diffusion MRI and appear to reduce the systematic errors incurred in mapping of axonal and cellular size in the living human brain. The SNR boost achieved on Connectome 2.0 enables visualization of small subcortical pathways and fibre configurations less than 2 mm in size, with a high degree of anatomical accuracy. The improvement in SNR paves the way toward mapping fine organizational principles of fibre pathways in individuals with neuropsychiatric disorders, thus opening avenues for personalized, image-guided intervention, for example, by neuromodulation of specific pathways.

Previous studies have shown that strong gradients sensitize the diffusion MRI signal to intra-axonal water diffusion and enable estimation of axon diameter index in the living human brain down to a diffusion resolution limit of 3–4 μm using gradients up to 300 mT m^−1^ (refs. ^24,27,33,69^). However, such measurements of effective axon diameter are weighted by the largest axons in the distribution and remain insensitive to smaller-diameter axons (~2–3 μm or less) that make up most white matter in the brain^27^. Moreover, estimation of effective axonal diameter was reproducible but noisy at the single-participant level on Connectome 1.0 (refs. ^46,70^). The enhanced SNR provided by the Connectome 2.0 gradient coil and high sensitivity receive arrays enables greater precision and consistency in estimation of tissue microstructural properties in white and grey matter (Supplementary Figs. 9 and 10). The boost in sensitivity and resolution provided by high-performance gradient systems may open doors for achieving precision human neuroscience and promises to provide powerful tools for probing variability in microscopic tissue organization and connectional anatomy, thereby increasing statistical power for understanding variation in brain structure and function at the individual level.

The large maximum gradient strengths achievable on Connectome 2.0 distinguish this system from other high-performance gradient systems, including the NexGen 7 T scanner equipped with the Impulse head gradient coil, which has lower *G*_max_ = 200 mT m^−1^ and higher SR_max_ = 900 T m^−1^ s^−1^, and was primarily designed to advance functional MRI studies at ultra-high spatial resolution^35^. Proposed 3 T head-only gradients with stronger gradients such as the MAGNUS 2.0 and NeuroFrontier scanners are also being realized with attainment of *G*_max_ ≥ 300 mT m^−1^ on par with Connectome 1.0 and slew rates ≥750 T m^−1^ s^−1^ (refs. ^37,71^). In particular, the very fast slew rates of these scanners will enable high-spatial-resolution imaging and a variety of diffusion-encoding paradigms across a variety of platforms, allowing ultra-high-performance gradients to permeate the realms of scientific and clinical research much more quickly. The engineering advances required to achieve such strong and fast gradients have broadly benefitted the radiological sciences and clinical imaging by encouraging the major scanner vendors to incorporate stronger and faster gradients into commercially available scanners^72^. Such strong gradients may enable MRI to monitor disease activity and pathologic changes at the microscopic level in real time without the inherent risks and biases of invasive sampling. Non-invasive tissue characterization with cellular-level specificity may offer earlier indicators of disease progression in a wide range of diseases, such as increased cellular density within the tumour treatment bed^73^, axonal and deep grey matter cellular loss in multiple sclerosis^74,75^, and alterations in cellular morphology in neurodegenerative disorders such as Alzheimer’s disease^7^. The engineering advances embedded in the Connectome 2.0 scanner and other ultra-high-performance scanners will enable additional classes of diffusion MRI measurements to be performed, offering a more sensitive probe of neural circuitry.

## Methods

The Connectome 2.0 scanner was built on a commercial 3 T magnet (MAGNETOM Vida, Siemens Healthiness, Erlangen, Germany) and integrated in the Siemens factory before delivery at the MGH. The design of the scanner began in 2018, with construction, testing and integration culminating in May 2023. The scanner was installed at MGH in July 2023 and has been fully operational since August 2023. All MRI data were acquired using Siemens Numarix X software v.XA61A.

### Gradient coil design

Candidate winding patterns were designed with a target-field method. In this approach a discretized continuous current density pattern is defined on a coil former mesh and optimized the participant to various design constraints including *B*_*z*_ field linearity, magnetic field amplitudes on the conductive magnet surfaces (active shielding), torque and force on the windings, inductance, and wire spacing and bend radii^36,76^. Coil windings were designed in an iterative fashion, alternating between conventional optimization with the target-field method and model-based PNS assessment to achieve the target specifications while attempting to maximize PNS thresholds and, therefore, the usable gradient- and image-encoding performance. The PNS prediction framework relies on detailed computational body models, with embedded atlases of the peripheral nervous system and a workflow combining electromagnetic and neurodynamic modelling^77^. The PNS prediction process starts by predicting the electric fields (E-fields) induced in the body by switching the gradient coil winding geometry. We then coupled these E-fields to an electric-circuit equivalent model of myelinated nerves to predict when and where the E-fields were expected to excite the peripheral nerves. The smallest coil current amplitude exciting any peripheral nerve in the body models determined the expected PNS threshold. This PNS model enabled assessment of the PNS characteristics of dozens of coil geometries and iterative refinement of the coil geometries to reduce PNS effects (raise PNS thresholds) without the need to build expensive prototype coils. The Connectome 2.0 design phase benefitted from the PNS insights obtained during the Impulse head coil’s design phase^35^ and incorporated information on how to balance PNS effects across different body regions (primarily the head and torso) to raise the worst-case PNS thresholds.

### PNS supervision

Standard PNS supervision was implemented on the system using gradient coil specific Stimulation Approximation by Filtering and Evaluation (SAFE) parameters^78^ determined in a volunteer study incorporating 29 participants and ensuring that the scanner complies with IEC standard 60601-2-33. Participants were positioned in the scanner with their shoulders touching the cutouts. The *z*-position of the participants was determined using the tune-up coil as a reference, such that participants were placed at isocentre inside the scanner. During the volunteer study, a test gradient pattern of 128 trapezoidal gradient pulses with a flat-top time of 500 µs and variable rise times between 50 µs and 3,000 µs was used. The gradient strength was increased in consecutive steps of 1 mT m^−1^ and the volunteers reported when they first felt PNS. The corresponding gradient strength is referred to as the stimulation threshold. This measurement was performed for each gradient axis (*X*, *Y*, *Z*) and the following worst-case combinations of different gradient axes (*X* ± *Y*, *Y* ± *Z*, *X* ± *Y* + *Z*). The averaged stimulation thresholds are shown in Fig. 3. We used logistic regression to estimate the population average stimulation values at each rise time.

### Gradient cooling system

A specialized cooling system was developed for the Connectome 2.0 gradient coil. This system directly cools a multifilament conductor using robust stainless-steel tubing to transport cooling water. A deliberate decision was made to utilize stainless-steel tubing due to its comparatively lower conductivity in relation to the copper filaments that envelop it. This attribute reduces the tubing’s sensitivity to eddy currents, which might otherwise disrupt the operation of the system. In addition, stainless steel possesses almost no magnetic properties, guaranteeing negligible interference with the homogeneity of the *B*_0_ magnetic field. Various design elements were incorporated into the gradient coil to facilitate efficient heat dissipation and sustain low temperatures. These included implementing large wire cross-sections, using parallel water circuits (>20) to minimize cooling loop lengths (<30 m), and the incorporation of high-pressure stainless-steel tubing to facilitate high flow rates (~40 l min^−1^) through the gradient coil. The combination of these elements facilitated the effective elimination of heat. The system is equipped with over 50 temperature sensors to continuously monitor the gradient coil’s temperature and ensure that the coil remains within safe operating temperature ranges, which is critical for maintaining the quality and reliability of MRI scans performed using the Connectome 2.0 gradient coil.

### Radiofrequency coils

The 72-channel in vivo coil consists of a single shell helmet to accommodate up to the 85th percentile of the adult male demographic (dimensions anterior–posterior: 200 mm, left–right: 180 mm, circumference at isocentre: 614 mm). The helmet surface was tiled with 72 loop coil elements, each approximately 60 mm in diameter, with 2 larger loops around the eyes. The 64-channel ex vivo coil consists of a coil former that is anatomically shaped to conform to an ex vivo brain specimen. The coil former can be opened and closed in the mid-section, allowing easy positioning of the ex vivo brain specimen within it. The coil former was designed to surround the brain sample and covered with 64 overlapped loop coil elements (diameter of 50 mm each). The Rx circuitry chain consisted of circular elements crafted from 1.5-mm-thick silver-plated wire. Each loop element was interconnected with a preamplifier daughterboard, which housed an on-board variable tuning capacitor for fine-tuning the loop’s resonance and a fine-tunable matching capacitor. A capacitive voltage divider at the port of the receive coil elements ensured symmetric RF current distribution along the loop element. For patient safety, the in vivo coil also incorporated a passive detuning circuit in the event of active detuning failure. Each coil element’s output was impedance-matched to 50 Ω. The same circuitry provided the necessary impedance transformation for accomplishing preamplifier decoupling^79^. Pairs of adjacent coil elements were connected to low-noise converter preamplifiers (Siemens Healthineers) that provided low-noise amplification and downconversion to two intermediate frequencies. These two signals were then multiplexed onto a single coaxial output. Each Rx coil array was equipped with its own dedicated local transmit (Tx) coil, designed as circularly polarized birdcage coils. Both transmit coils share the same electrical layout but utilize different matching networks to address the differing load characteristics of a human head and an ex vivo brain specimen. The specific parameters of the transmit coils are as follows: a 16-rung hybrid birdcage design with a coil diameter of 316 mm and a coil length of 236 mm. Each local transmit coil incorporated its own RF shield consisting of thin slotted copper, with a shield diameter of 363 mm and a shield length of 280 mm. For mitigating eddy currents on the RF screen, the shield was constructed from 9-μm-thick slotted copper, which provided poor conductivity in terms of RF skin depth for eddy currents but maintained a well-conditioned skin depth for the birdcage’s RF mirror currents. Similarly, the RF shield on the inner surface of the gradient coil (44 cm diameter) also consisted of thin slotted copper. The birdcage coils were tuned to their primary resonant frequency at 123.25 MHz through end-ring capacitors (43 pF each). To set the birdcage coils into an actively tuned state, 2 PIN diodes were centrally integrated into each rung. They were forward biased by 125 mA during the Tx pulsing period.

Assessing the SNR performance of the constructed coils, we compared the 72-channel head coil with a standard 32-channel in vivo head coil, the most commonly used head coil on the Connectome 1.0 scanner, and a dedicated 64-channel in vivo head coil constructed for the MGH original Connectome 1.0 scanner^42^ using an anthropomorphic head phantom. The 64-channel ex vivo brain coil was directly compared to the 72-channel head coil using an agar brain phantom. Pixel-wise SNR maps^80^ were calculated for images formed from the noise- and covariance-weighted sum of squares of the individual channel images acquired with a proton density-weighted FLASH sequence. Parameters were: TR/TE = 200/4.8 ms, flip angle (FA) = 15°, matrix size: 192 × 192, FOV: 256 mm × 256 mm, slice thickness: 6 mm, bandwidth (BW): 200 Hz per pixel, number of averages = 6. Noise covariance information was acquired using the same pulse sequence but without RF excitation. Facilitating direct comparison of the receive arrays with different transmit coils, the SNR maps were normalized by the spatially corresponding flip angle maps, which were obtained using the double-angle method^81,82^. The receiver array’s encoding capabilities for undersampled data acquisitions were assessed using inverse g-factor maps^83^ for representative slices in the phantom.

The design and construction of a receiver coil array with 72 channels for the Connectome 2.0 scanner was a deliberate decision to optimize SNR, especially accelerated SNR. This choice was driven by practical considerations that ensured sample noise dominance for each coil detector element. An unloaded-to-loaded *Q*-ratio of 2 indicates equal noise distribution between sample and electronics, while a *Q*-ratio of 3 or higher is desired to ensure that sample noise dominates throughout the coil array. The 64-channel Connectome 1.0 head coil had an unloaded-to-loaded *Q*-ratio of 4 (ref. ^42^), allowing for slightly smaller loop elements with a correspondingly lower *Q*-ratio. Our 72-channel coil met these stringent criteria, achieving an average *Q*-ratio of 3.1, while array configurations exceeding 72 channels did not meet the required specifications. This balance ensured that SNR was maximized without compromising coil system performance due to resistive electronic losses. The 72-channel head coil also provided slightly improved g-factors in direct comparison to the 64-channel coil, thus enhancing accelerated SNR.

A practical limitation of the in vivo coil design was a smaller helmet size. This choice was influenced by the critical radial space constraints imposed by the gradient coil and the necessity for optimal spatial separation of the volume transmitter coil structure, the 72-channel receiver coil array, and the 16-channel field camera system to prevent mutual interference and preserve RF performance integrity. Although the 72-channel in vivo array coil is below the industry standard of approximately the 95th percentile, it was essential to ensure high functionality for our specific research application. Therefore, sizing at the 85th percentile was determined to be the most practical solution, striking a balance between accommodating a substantial portion of the population (covering nearly 100% of the female and 85% of the male population) and maintaining the RF performance integrity of the coil ensemble.

The implementation strategy of integrating the 16-channel dynamic field monitoring system into each receiver coil array was constrained by several critical factors. Notably, the limited radial space within the gradient coil bore precluded the addition of commonly used clip-on or shell-mounted field camera systems. This necessitated the integration of the field camera into the coil housing. In addition, the field camera probes required positioning close to the participant’s head to remain within the 20-cm-diameter gradient linearity zone, which is critical for accurate field assessment of the brain region.

The integration process also emphasized preventing mutual interference between the Tx coil, Rx coil array and field camera. Careful component management around the helmet ensured that all components operated harmoniously without compromising the RF signal integrity of each coil system. A particularly notable challenge was maintaining a 2 cm clearance between the field camera probes and surrounding materials, including plastic, electronics, cables and circuit boards, all contained within a densely packed coil housing. This clearance was critical to prevent susceptibility modulation within the signal pick-up region of the camera probes, which could substantially shorten the field probe free induction decay lifetime. The overall helmet’s component packaging needed to ensure that the 16 camera probes provided consistently orthogonal signal content. This orthogonality is essential for uniquely identifying and accurately reconstructing each spatial harmonic component up to the 3rd order. Moreover, the workflow efficiency between in vivo and ex vivo scan sessions was substantially enhanced by incorporating the field monitoring system into each coil array, facilitating quick and seamless coil changes on the MRI patient table.

### Concurrent field monitoring

A healthy adult volunteer (23-year-old female) was scanned on the Connectome 2.0 scanner using the custom-built 72-channel head coil, with written informed consent and ethics approval in place. Whole-brain diffusion MRI was acquired with a two-dimensional (2D) monopolar pulsed gradient spin echo (PGSE) EPI sequence with 100 axial slices, 0.9 mm in-plane resolution, slice thickness = 0.9 mm, TR/TE = 12,000/47 ms, phase-encoding direction anterior to posterior, in-plane acceleration factor = 2, partial Fourier (PF) = 6/8, no simultaneous multislice (SMS), BW = 1,912 Hz per pixel and echo spacing = 0.57 ms. Total acquisition time was 7.2 min. DWIs (30) were acquired with isotropically distributed diffusion-encoding gradient directions at *b* = 1,200 s mm^−2^ (*G*_max_ = 500 mT m^−1^, *Δ* = 8.7 ms, *δ* = 2.9 ms). A non-diffusion-weighted image (*b* = 0) was also acquired. A gradient-recalled echo scan was performed after the diffusion scan to estimate the coil sensitivity profile. The 2D-PGSE-EPI sequence was modified by incorporating triggers and synchronization pulses at the beginning of the sequence to ensure accurate synchronization between the monitored field and the *k*-space data acquired during the image readout^40^. A 3rd-order spherical harmonics model was fitted to the phase of the signal for each of the 16 ^19^F MR probes with a linear least-squares algorithm^45^. *B*_0_-eddy-current compensation applied by the scanner was removed before reconstruction^45,84^. Images were reconstructed with a modified version of the SENSitivity Encoding (SENSE) method^83^ implemented in MATLAB R2023b code, with the image-encoding matrix informed by the phase evolution^45,49^. The efficient iterative self-consistent parallel imaging reconstruction (ESPIRiT) algorithm was used to estimate the coil sensitivities^85^. The magnitude of the reconstructed complex images was used for analysis and visualization. Concurrent field monitoring-based image reconstruction was compared against standard GRAPPA^86^ reconstruction with one-dimensional navigators for Nyquist ghosting reduction based on a linear phase model^50^ and against dual-polarity GRAPPA^51^. Online reconstruction was used. Reconstructed images with GRAPPA and dual-polarity GRAPPA were corrected for eddy current-induced geometric distortion with the post-processing tool ‘eddy’^87^ (FMRIB Software Library, Oxford, United Kingdom).

### SNR performance comparisons

Diffusion MRI measurements were performed on a healthy adult volunteer (43-year-old female), with written informed consent and ethics approval in place. All measurements were performed with a 2D monopolar PGSE-EPI sequence^8,88^ on the Connectome 2.0 scanner with the 72-channel in vivo head coil. To evaluate the SNR performance on the basis of the gradient parameters of the Connectome 2.0, Connectome 1.0 and clinical scanner, we acquired DWIs on the Connectome 2.0 scanner using three different protocols with a fixed pulse width of 8 ms and the following diffusion-weighting *b*-values, diffusion times *Δ* and TE:

- Connectome 2.0 scanner protocol (*G*_max_ = 500 mT m^−1^, SR_max_ = 600 T m^−1^ s^−1^): (*b*, *Δ*, TE) = (5,000 s mm^−2^, 15 ms, 40 ms), (10,000 s mm^−2^, 15 ms, 40 ms), (20,000 s mm^−2^, 23 ms, 48 ms), (30,000 s mm^−2^, 32.2 ms, 57 ms), (40,000 s mm^−2^, 39.8 ms, 65 ms)
- Connectome 1.0 scanner protocol (*G*_max_ = 300 mT m^−1^, SR_max_ = 80 T m^−1^ s^−1^): (*b*, *Δ*, TE) = (5,000 s mm^−2^, 17.9 ms, 46 ms), (10,000 s mm^−2^, 27.2 ms, 55 ms), (20,000 s mm^−2^, 51.5 ms, 79 ms), (30,000 s mm^−2^, 76 ms, 106 ms), (40,000 s mm^−2^, 100 ms, 128 ms).
- State-of-the-art clinical scanner protocol (*G*_max_ = 80 mT m^−1^, SR_max_ = 80 T m^−1^ s^−1^): (*b*, *Δ*, TE) = (5,000 s mm^−2^, 150 ms, 176 ms)

The maximum achievable slew rates for Connectome 1.0 and the state-of-the-art clinical scanner are derated to 80 T m^−1^ s^−1^ to avoid cardiac nerve stimulation^17^. For each scan, we obtained 15 *b* = 0 images and DWIs in 64 diffusion gradient directions, with slice thickness of 2 mm, FOV of 220 mm × 220 mm, and matrix size of 110 × 110. The whole brain volume was scanned using 79 axial slices. All scans were performed with the same TR = 3,800 ms and echo spacing = 0.42 ms. Other relevant parameters were in-plane acceleration factor = 2 and PF = 6/8. The SNR performance of each (*b*, *Δ*, TE) combination was evaluated in the cerebral white matter on the basis of the temporal SNR of the last 10 *b* = 0 images, which were acquired consecutively at the end of each scan. The white matter mask was generated using SynthSeg (Freesurfer)^89^. The theoretical gain in SNR with respect to the Connectome 1.0 protocol was calculated on the basis of mono-exponential *T*_2_-relaxation ~exp{−[TE(*b*)–TE_*C*1_(*b*)]/*T*_2_}, where TE_*C*1_ is the echo time of the Connectome 1.0 protocol, and *T*_2_ = 80 ms is the transverse relaxation time in white matter at 3 T (ref. ^90^).

### Theoretical prediction of the shortest echo time

We built a PGSE-EPI sequence simulator to calculate the theoretical value of the shortest echo time (TE) on the basis of other sequence parameters, including maximal gradient strength *G*_max_, maximal slew rate SR_max_, diffusion time *Δ* and pulse width *δ* of diffusion gradients, *b*-value, the duration of the 90° RF excitation pulse (*t*_90_ = 2.1 ms), the duration of reference lines (*t*_REF_ = 1.76 ms) right after excitation pulse for Nyquist ghost correction, and 180° RF refocusing pulse (*t*_180_ = 3.4 ms), image matrix size in the phase-encoding direction *N*, PF factor, GRAPPA acceleration factor R_GRAPPA_, echo spacing ESP, and the time to travel from the centre of the *k*-space to the beginning of the EPI trajectory (*t*_*k*0-EPI_ = 0.5 ms). The duration between the beginning of the EPI and the signal echo was *t*_EPI−echo_ = *N*(PF−1/2)/*R*_GRAPPA_ × ESP + *t*_*k*0−EPI_. For a fixed value of *δ*, the shortest possible TE was optimized as follows:

1. First, we calculated the lower bound for TE, defined as TE_lb_ = *t*_180_ + 2(*δ* + *G*_max_/SR_max_ + *t*_EPI−echo_), and positioned (1) the first diffusion gradient right after the excitation pulse and reference lines and (2) the second diffusion gradient right after the refocusing pulse. If the resulting *b*-value was larger than the targeted *b*-value, we set the TE = TE_lb_; otherwise, we further optimized the TE in the second step.
2. Second, we positioned (1) the first diffusion gradient right after the excitation pulse and reference lines and (2) the second diffusion gradient right before the imaging gradient and expressed the *b*-value as a function of TE. The resulting *b*-value monotonically increased with the TE. Thus, for a given *b*-value, we could set the shortest TE required to accommodate the RF pulses, diffusion gradients and imaging gradients.

### High-spatial-resolution diffusion tractography

Diffusion measurements were carried out on a healthy adult volunteer (34-year-old female) using Connectome 2.0 and 1.0 diffusion tractography protocols on the Connectome 2.0 scanner using the 72-channel in vivo head coil. Ethics approval was obtained before the experiment. The participant also provided informed consent. For both protocols, DWIs were acquired using a 2D monopolar PGSE-EPI sequence with a voxel size of 1 mm at 2 *b*-values: *b* = 1,000 and 2,500 s mm^−2^ with 64 diffusion gradient directions each. Interspersed *b* = 0 images were acquired every 16 DWIs. An additional *b* = 0 image with reverse phase-encoding direction (left to right) was obtained to correct susceptibility-induced distortions. Other parameters included FOV = 180 mm × 180 mm, matrix size = 180 × 180, 132 slices, slice thickness = 1 mm, phase-encoding direction = right to left, in-plane acceleration factor = 2, PF = 6/8, no SMS, and BW = 1,984 Hz per pixel. For each protocol, the diffusion time *Δ* and gradient pulse width *δ* were adjusted to achieve the shortest possible TE within the limits of *G*_max_ and SR_max_. Relevant parameters of the Connectome 2.0 protocol were *G*_max_ = 500 mT m^−1^, SR_max_ = 600 mT m^−1^, *Δ* = 13.6 ms, *δ* = 3.5 ms, TR/TE = 12,000/40 ms. The total scan time was 26 min. Relevant parameters of the Connectome 1.0 protocol were *G*_max_ = 300 mT m^−1^, SR_max_ = 200 mT m^−1^, *Δ* = 24.7 ms, *δ* = 4.2 ms and TR/TE = 17,400/67 ms. The total scan time was 37 min. The preprocessing pipeline for both protocols was based on the DESIGNER pipeline^91^. This included iterative Rician-corrected denoising^92,93^, Gibbs ringing correction^94^, susceptibility and eddy current-induced distortion correction^87,95^, and gradient nonlinearity distortion correction^96,97^. We fitted fibre orientation distribution functions (fODFs) to the preprocessed data using a multishell, multitissue constrained spherical deconvolution algorithm in MRtrix3 (ref. ^98^). Whole-brain tractograms were then obtained by seeding local probabilistic tractography^99^ in every voxel within a white matter mask (5 seeds per voxel). Other tractography parameters included step size = 0.5 mm, angle threshold = 45° and maximal order of spherical harmonics *l*_max_ = 8. To allow for better anatomical comparison, we co-registered the two datasets using a highly accurate inverse registration approach^100^ and applied the linear transformations to the fODFs volumes and tractography outputs.

### In vivo human brain tissue microstructure

Diffusion measurements were performed in 10 healthy adult participants (31.0 ± 6.9 years, 5 females) on the Connectome 2.0 scanner using the 72-channel head coil and compared against comparable diffusion MRI data acquired in 10 age- and sex-matched participants (31.7 ± 7.0 years, 5 females) on the Connectome 1.0 scanner^101^ using a 64-channel head coil^42^. All participants provided informed consent. Ethics approval was obtained before the study. For both protocols, we acquired DWIs using a 2D monopolar PGSE-EPI sequence with isotropic voxel size of 2 mm. Other parameters in common included: FOV = 220 mm × 220 mm, 66 slices, slice thickness = 2 mm, phase-encoding direction = anterior to posterior, in-plane acceleration factor = 2, PF = 6/8, SMS = 2 and BW = 2,840 Hz per pixel. On the Connectome 2.0 scanner, we acquired DWIs at 8 *b*-values: *b* = 50, 350, 800, 1,500, 2,400, 3,450, 4,750 and 6,000 s mm^−2^ at *Δ* = 13 ms, and another set of 8 *b*-values: *b* = 200, 950, 2,300, 4,250, 6,750, 9,850, 13,500 and 17,800 s mm^−2^ at *Δ* = 30 ms. Other parameters were *δ* = 6 ms, TR/TE = 3,600/53 ms, *G*_max_ = 500 mT m^−1^ and SR_max_ = 600 mT m^−1^. On the Connectome 1.0 scanner, we acquired DWIs with the same 2 sets of *b*-values but with diffusion times *Δ* = 19 ms and *Δ* = 49 ms, respectively, based on the available *G*_max_ and SR_max_. Other parameters were *δ* = 8 ms, TR/TE = 3,800/77 ms, *G*_max_ = 300 mT m^−1^ and SR_max_ = 80 mT m^−1^. For both Connectome 2.0 and 1.0 scanners, an interspersed *b* = 0 image was acquired every 16 DWIs. An additional *b* = 0 image acquired with reversed phase-encoding direction (posterior to anterior) was obtained to correct for susceptibility-induced distortions. For lower *b*-values (*b* ≤ 2,300 s mm^−2^), DWIs were acquired with 32 diffusion gradient directions homogeneously distributed over a unit sphere; for higher *b*-values (*b* > 2,300 s mm^−2^), DWIs were acquired with 64 gradient directions. The DWIs were preprocessed on the basis of the DESIGNER pipeline^91^, including Gibbs ringing correction^94^, susceptibility and eddy current-induced distortion correction^87,95^, gradient nonlinearity distortion correction^96,97^ and Rician bias correction^102^.

### Biophysical modelling

For each *b*-value, the diffusion signals were averaged over all directions to factor out fibre orientation dispersion, yielding the so-called spherical mean signals^103,104^. For axonal diameter mapping in the cerebral white matter, the AxCaliber-SMT model^21^ was fitted to the spherical mean signals of the 16 *b*-values acquired on Connectome 2.0 (*Δ* = 13, 30 ms) and Connectome 1.0 (*Δ* = 19, 49 ms) scanners. Voxel-wise fitting was performed using a Markov chain Monte Carlo sampling approach to yield estimates of apparent axonal diameter, restricted volume fraction, free water volume fraction and hindered diffusivity. The longitudinal diffusivity in intra- and extracellular spaces was fixed at 1.7 µm^2^ ms^−1^.

To generate averaged axonal diameter maps for comparison between the two scanners, axonal diameter maps were first transformed from each individual’s native diffusion space to the common MNI152 space and then averaged across individuals scanned on Connectome 2.0 and Connectome 1.0 scanners separately. The warps used for this transformation were generated by registering the individual FA maps to the HCP-1065-FA template^105^ using the nonlinear registration tool ‘fsl_reg’ (FMRIB Software Library)^106^. To compare the distribution of axon diameter indices obtained on the two scanners, we calculated and plotted the histogram of axon diameter indices extracted from voxels in a representative region of interest (ROI) within the posterior corona radiata obtained from the Johns Hopkins University (JHU) white matter probabilistic tractography atlas^107^. Furthermore, for each scanner, we averaged axon diameter indices over the 10 participants in other ROIs from the JHU atlas, including the corpus callosum, anterior and posterior limbs of the internal capsule, and anterior corona radiata. We compared the results between the two scanners using an unpaired-samples *t*-test, corrected for multiple comparisons using the FDR.

For soma size estimation in the grey matter, the SANDI model^30^ was fitted to the spherical mean signals of the 8 *b*-values acquired at the shorter diffusion times on Connectome 2.0 (*Δ* = 13 ms) and Connectome 1.0 (*Δ* = 19 ms) separately, to avoid the effects of intercompartmental water exchange at longer time scales (>20 ms). We performed voxel-wise fitting using a random forest regression algorithm and estimated the apparent soma radius, intrasoma signal fraction, intraneurite diffusivity, intraneurite signal fraction and extracellular diffusivity. The SANDI metrics were projected onto the FreeSurfer-averaged inflated cortical surface^108,109^ and averaged across 10 participants scanned on each scanner, labelled with sensorimotor cortex encompassing primary motor cortex (Brodmann area 4) and somatosensory cortex (Brodmann areas 3a, 3b and 1). Intrasoma signal fraction was compared among the subregions of the sensorimotor cortex using a paired-samples *t*-test with FDR correction applied for the multiple comparisons. Similarly, soma radius was compared between the two scanners using an unpaired-samples *t*-test.

### Reporting summary

Further information on research design is available in the Nature Portfolio Reporting Summary linked to this article.

## Supplementary information

Supplementary Information Supplementary Figs. 1–10, Table 1 and Note 1.

Reporting Summary

## Data Availability

Raw and preprocessed diffusion-weighted images used to estimate axon diameter are publicly available on OpenNeuro at https://openneuro.org/datasets/ds006181/versions/1.0.0 (ref. ^110^). All other data are available from the corresponding authors upon reasonable request.

## Code availability

Code for theoretical prediction of shortest echo time (TE) is publicly available on Zenodo^111^ and on GitHub at https://github.com/Connectome20/protocol_design_PGSE. AxCaliber-SMT code is publicly available on Zenodo^112^ and on GitHub at https://github.com/Connectome20/Biophysical-modeling. Other image reconstruction, processing and analysis code is available from the corresponding authors upon reasonable request.
