# Publisher Correction: Ultra-high gradient connectomics and microstructure MRI scanner for imaging of human brain circuits across scales

**DOI:** 10.1038/s41551-025-01484-8

**Published:** 2025-07-22

**Authors:** Gabriel Ramos-Llordén, Hong-Hsi Lee, Mathias Davids, Peter Dietz, Andreas Krug, John E. Kirsch, Mirsad Mahmutovic, Alina Müller, Yixin Ma, Hansol Lee, Chiara Maffei, Anastasia Yendiki, Berkin Bilgic, Daniel J. Park, Qiyuan Tian, Bryan Clifford, Wei-Ching Lo, Stefan Stocker, Jasmine Fischer, Gudrun Ruyters, Manuela Roesler, Andreas Potthast, Thomas Benner, Elmar Rummert, Rebecca Schuster, Peter J. Basser, Thomas Witzel, Lawrence L. Wald, Bruce R. Rosen, Boris Keil, Susie Y. Huang

**Affiliations:** 1 Athinoula A. Martinos Center for Biomedical Imaging, Department of Radiology, Massachusetts General Hospital, Charlestown, MA USA; 2 Harvard Medical School, Boston, MA USA; 3 Siemens Healthineers, Erlangen, Germany; 4 Institute of Medical Physics and Radiation Protection, TH-Mittelhessen University of Applied Sciences, Giessen, Germany; 5 Center for Neurotechnology and Neurorecovery, Department of Neurology, Massachusetts General Hospital, Boston, MA USA; 6 Siemens Medical Solutions USA, Boston, MA USA; 7 Eunice Kennedy Shriver National Institute of Child Health and Human Development, National Institutes of Health, Bethesda, MD USA; 8 Q Bio Inc., San Carlos, CA USA; 9 Department of Diagnostic and Interventional Radiology, University Hospital Marburg, Philipps University of Marburg, Marburg, Germany; 10 LOEWE Research Cluster for Advanced Medical Physics in Imaging and Therapy (ADMIT), TH-Mittelhessen University of Applied Sciences, Giessen, Germany

**Keywords:** Imaging techniques, Magnetic resonance imaging

Correction to: *Nature Biomedical Engineering* 10.1038/s41551-025-01457-x, published online 16 July 2025.

In the version of this article originally published, due to a conversion error, images of brains were missing in Fig. 6a. The figure is now corrected in the HTML and PDF versions of the article.

