# Supplementary material for: Ultra-high gradient connectomics and microstructure MRI scanner for imaging of human brain circuits across scales
Source: Nat Biomed Eng. 2025 Jul 16;10(2):309–24. doi: 10.1038/s41551-025-01457-x (PMC12920100; doi:10.1038/s41551-025-01457-x)
Supplement: Supplementary file 1 — Supplementary Figs. 1–10, Table 1 and Note 1. [file 41551_2025_1457_MOESM1_ESM.pdf]

nature biomedical engineering

Supplementary information https://doi.org/10.1038/s41551-025-01457-x

Ultra-high gradient connectomics and microstructure MRI scanner for imaging of human brain circuits across scales

In the format provided by the authors and unedited

This PDF file includes:

Supplementary Figures

Supplementary Fig. S1. Comparison of SNR performance of representative protocols on the Connectome 2.0 scanner and different B_0 field.

Supplementary Fig. S2. Results of gradient nonlinearity correction.

Supplementary Fig. S3. Gradient response function (GIRF) of the Connectome 2.0 gradient system.

Supplementary Fig. S4. High-order GIRF analysis of Connectome 2.0 gradient system**.**

Supplementary Fig. S5. Comparison of the Connectome 2.0 gradient experimental PNS thresholds (red) with other head gradient coils. Solid lines show PNS thresholds and dashed lines show hardware operational regions (determined by G_{max}/S_{max}).

Supplementary Fig. S6. Comparison of the 72-channel in vivo coil with a 64-channel in vivo coil constructed for the MGH Connectome 1.0 scanner.

Supplementary Fig. S7. Concurrent field monitoring.

Supplementary Fig. S8. Non-linear Nyquist ghosting removal.

Supplementary Fig. S9. Mean and standard deviation of mean axon diameter index across representative white matter regions of interest.

Supplementary Fig. S10. Intra-soma signal fraction in subregions of sensorimotor cortex.

Supplementary Fig. S11. The multiparametric MRI obtained using the Connectome 2.0 scanner.

Supplementary Tables

Supplementary Table S1. Summary of experimental PNS threshold characteristics measured for the Connectome 2.0 head gradient.

Supplementary Notes

Supplementary Note S1. Acquisition details of multiparametric MRI obtained with the Connectome 2.0 scanner

Supplementary References

Supplementary Figures

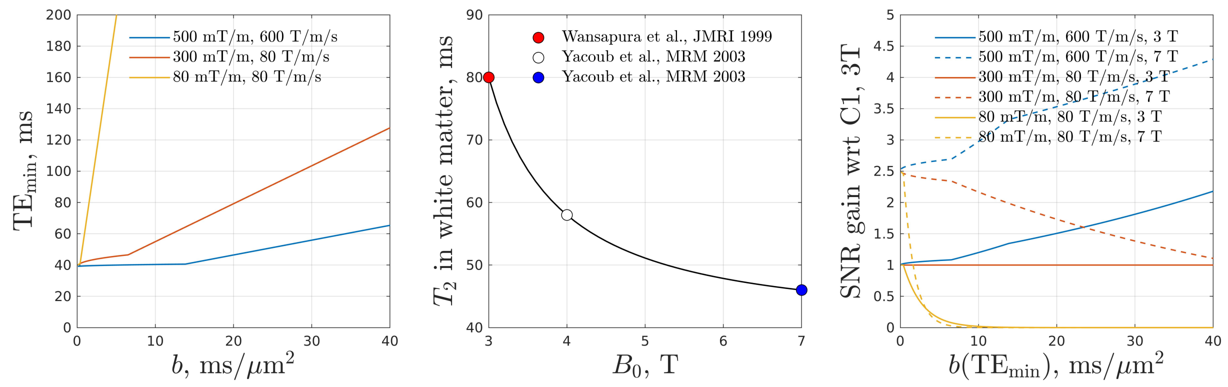

Supplementary Fig. S1 | Comparison of SNR performance of representative protocols on the Connectome 2.0 scanner and different B_0 field. (Left) Compared with the Connectome 1.0 protocol at 3T (red), the minimal TE required to achieve a given b-value is shorter when using the Connectome 2.0 protocol (blue). (Middle) Based on the literature, in the brain white matter, the lower the B_0, the longer the T_2 values. (Right). The TE shortening enabled by the Connectome 2.0 gradients yields an SNR gain of ~1.2 to 2 compared to the Connectome 1.0 protocol at the highest b-values. To estimate the SNR gain due to increasing field strength, we approximate the human brain as a dielectric sphere in radius of 7 cm [1] with dielectric properties of brain tissues, leading to an ultimate intrinsic SNR gain of 3.6 on a 7T scanner with respect to 3T ones [2]. However, the greater T2-relaxation-induced signal loss at 7T reduces this gain. For instance, a 7T MRI scanner with G_{max} = 500 mT/m and SR_{max} = 600 T/m/s could yield an SNR gain of up to 2× over the Connectome 2.0 scanner for high b-value diffusion imaging. The curves represent the theoretical predictions using a mono-exponential decay (assuming T_2=80 ms in white matter at 3T and T_2=46 ms at 7T based on previous literature).

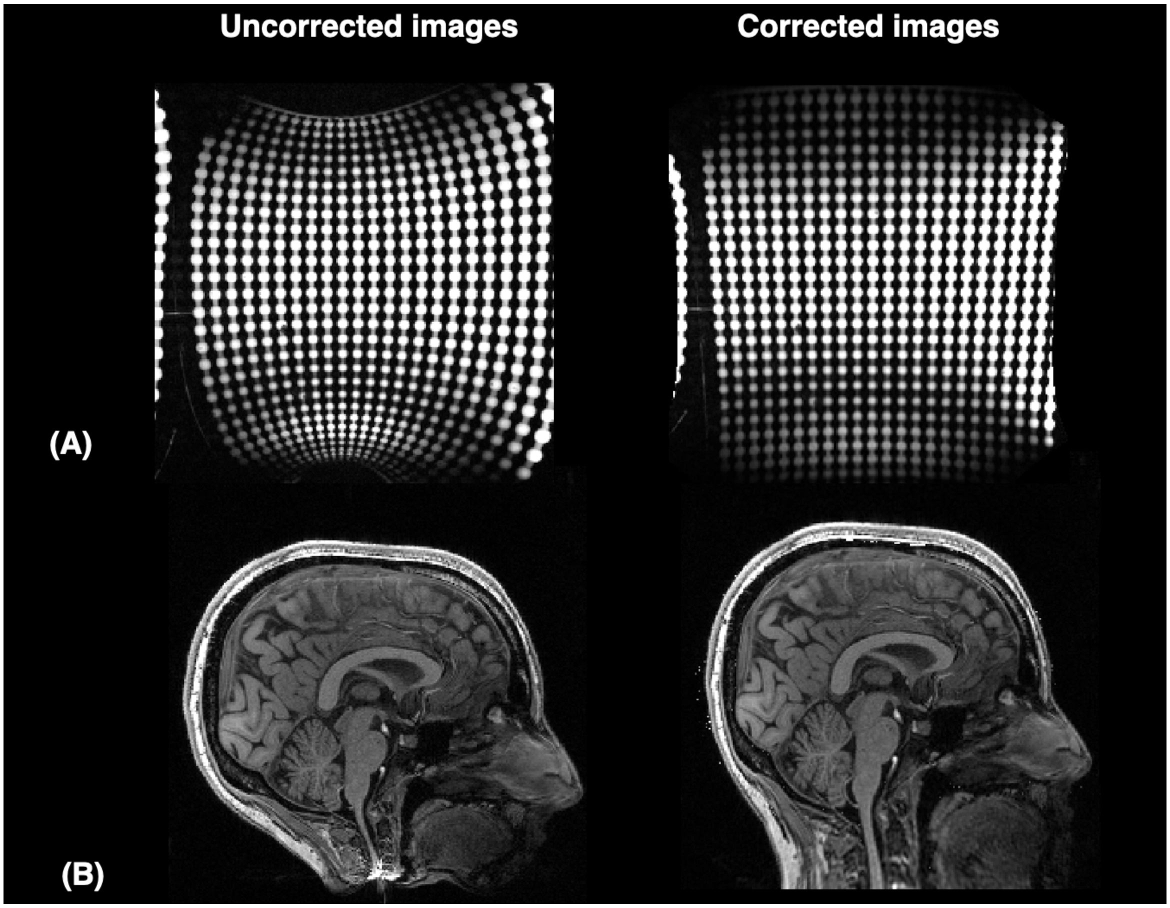

Supplementary Fig. S2 | Results of gradient nonlinearity correction. (a) Axial view of a grid phantom image without (left) and with (right) gradient nonlinearity correction, **(b)** Sagittal view of a high-resolution T1-weighted image of a human brain without (left) and with (right) gradient nonlinearity correction.

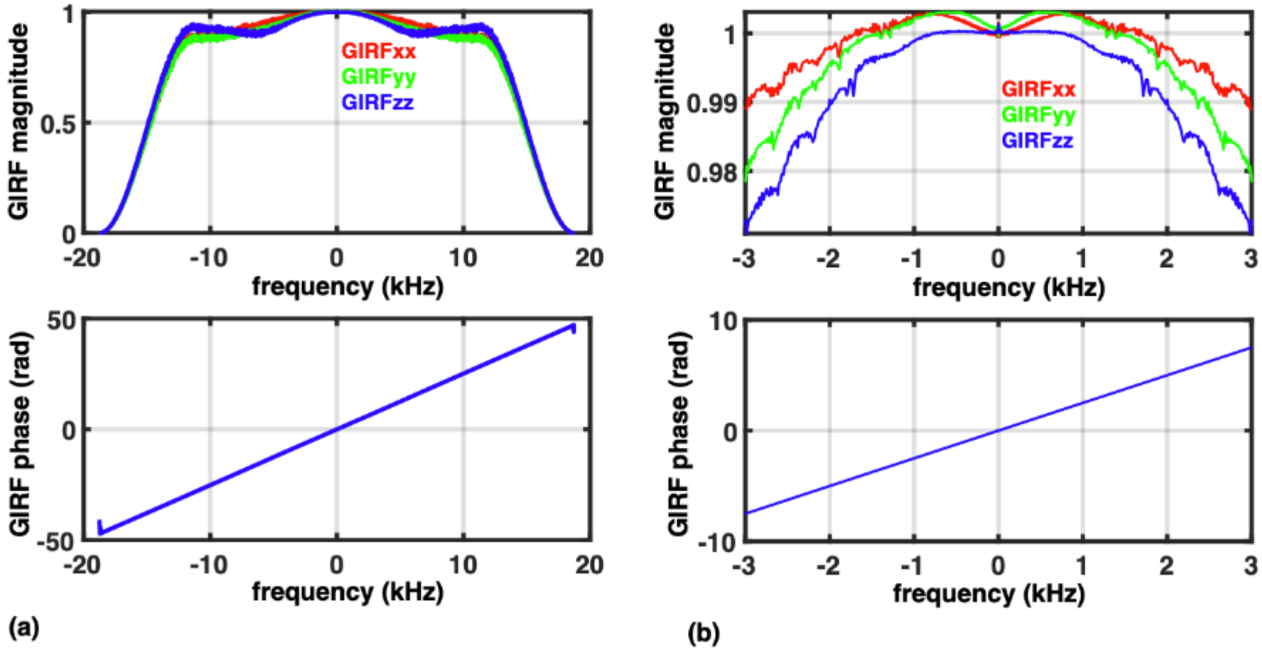

Supplementary Fig. S3 l Gradient response function (GIRF) of the Connectome 2.0 gradient system. **a,** Magnitude and phase of measured GIRFs with built-in eddy current compensation for all three gradient directions. The low pass characteristic of the gradient system is clearly visible in **(a)**. Details on the response in **(b)** demonstrate the distinct behavior for each gradient direction.

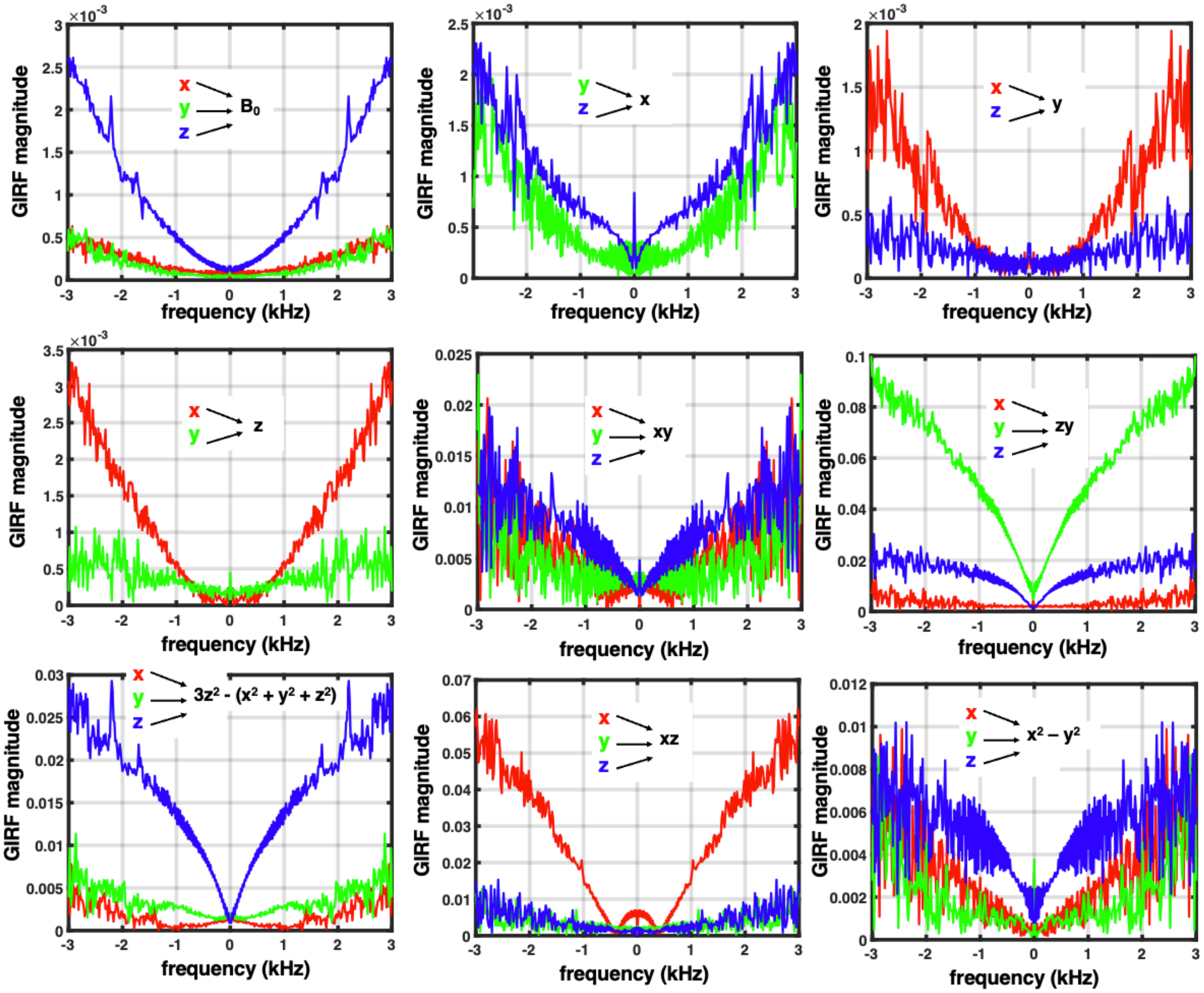

Supplementary Fig. S4 | High order GIRF analysis of the Connectome 2.0 gradient system. Magnitude of the measured cross-responses of the $B_0,\ x,\ y,\ z,\ xy,\ zy,\ 3z^2-(x^2+y^2+z^2),\ xz,\ x^2-y^2$ spherical harmonic field terms due to operating the x- (red), y- (green), and z-gradients (blue).

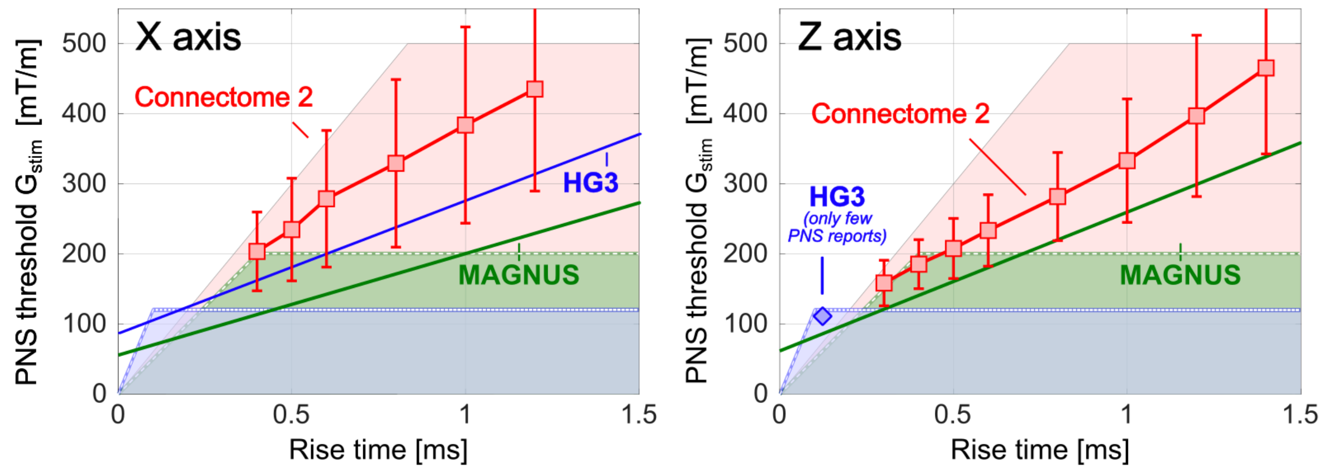

Gradient system	Diameter ROL	Field deviation	G_{max} [mT/m] / S_{max} [T/m/s]	Inner diameter	Inductance
Connectome 2 (red line, square)	20 cm	**6.7 to 11.7%**	500 / 600	44 cm	1800 − 2250 µH
MAGNUS (green line)	26 cm	**18%**	200 / 500	42 cm	710 − 920 µH
HG3 (blue line, diamond)	19–22 cm	—	120 / 1200	—	—

Supplementary Fig. S5 I Comparison of the Connectome 2.0 gradient experimental PNS thresholds (red) with other head gradient coils. Solid lines show PNS thresholds and dashed lines show hardware operational regions (determined by G_{max}/S_{max}). The PNS experiments of the Connectome 2 coil used 0.5 ms flat-top waveforms, while the “MAGNUS” [3] and “HG3” [4] used 1 ms flat top, which is known to yield similar thresholds [5,6].

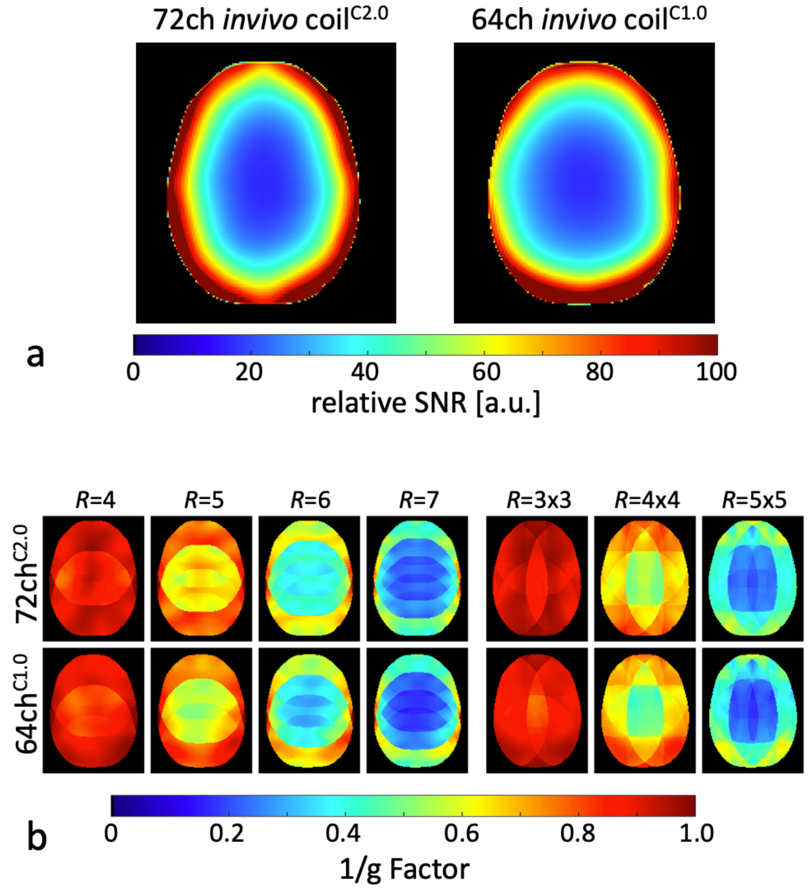

Supplementary Fig. S6 | Comparison of the 72-channel in vivo coil with a 64-channel in vivo coil constructed for the MGH Connectome 1.0 scanner. a. SNR maps comparison. **b.** g-factor maps comparison.

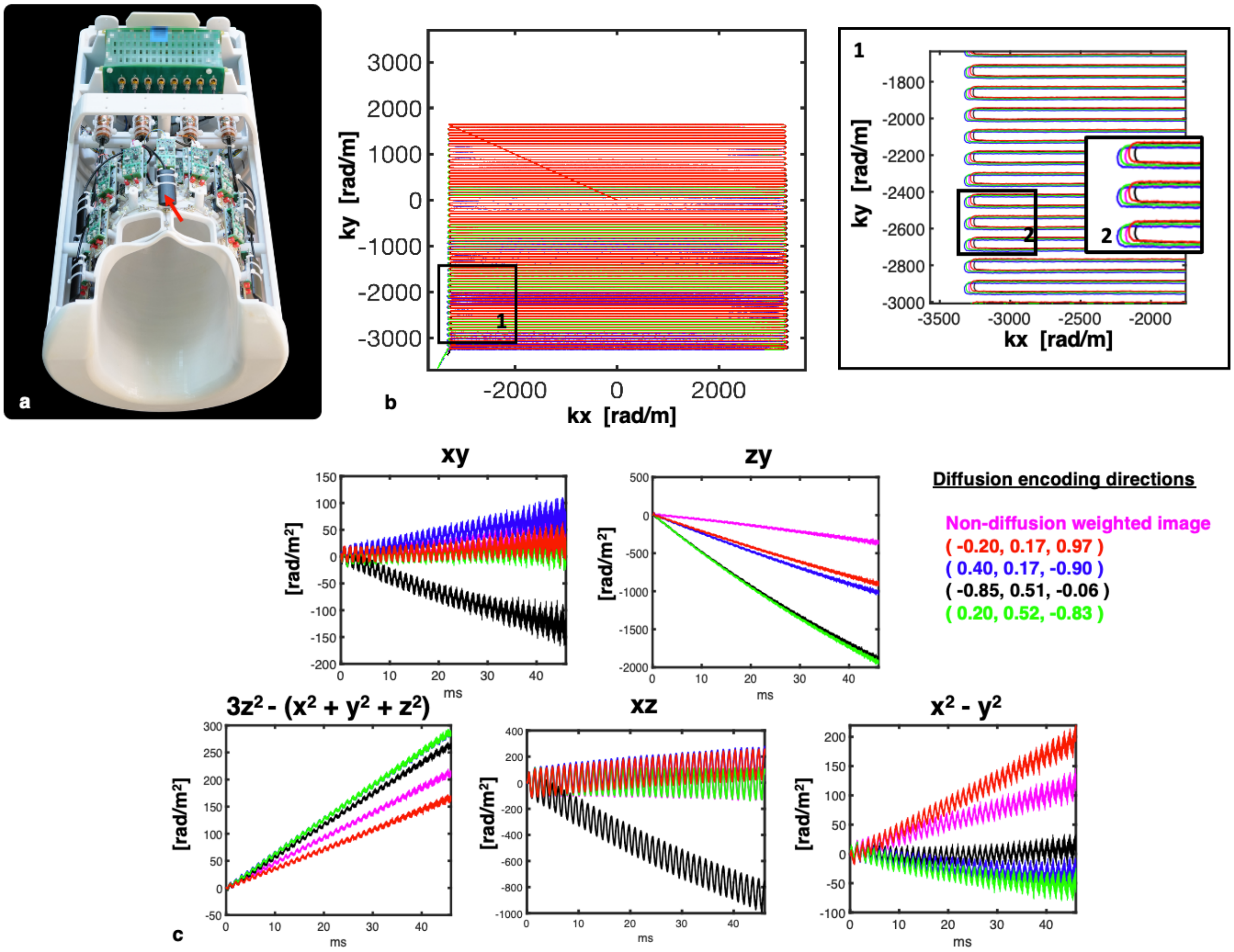

Supplementary Fig. S7 | Concurrent field monitoring. a, 16-channel dynamic field monitoring system consisting of ^{19}F field probes (red arrow) integrated in the 72-channel *in vivo* coil. **b**, Measured imaging *k*-space trajectory of a non-diffusion weighted (*b*=0) image (magenta) and four representative DWIs acquired at *b* =1,200 s/mm^2 (G_{max}=500 mT/m, Δ=8.7 ms, δ=2.9 ms) with different diffusion directions at 0.9 mm isotropic resolution. Note that the *k*-space trajectories deviate from the nominal prescribed *k*-space trajectory (magenta) due to residual eddy currents from the diffusion gradients. **c**, Temporal evolution of 2nd order eddy current phase terms measured during image readout with the 16-channel dynamic field monitoring system.

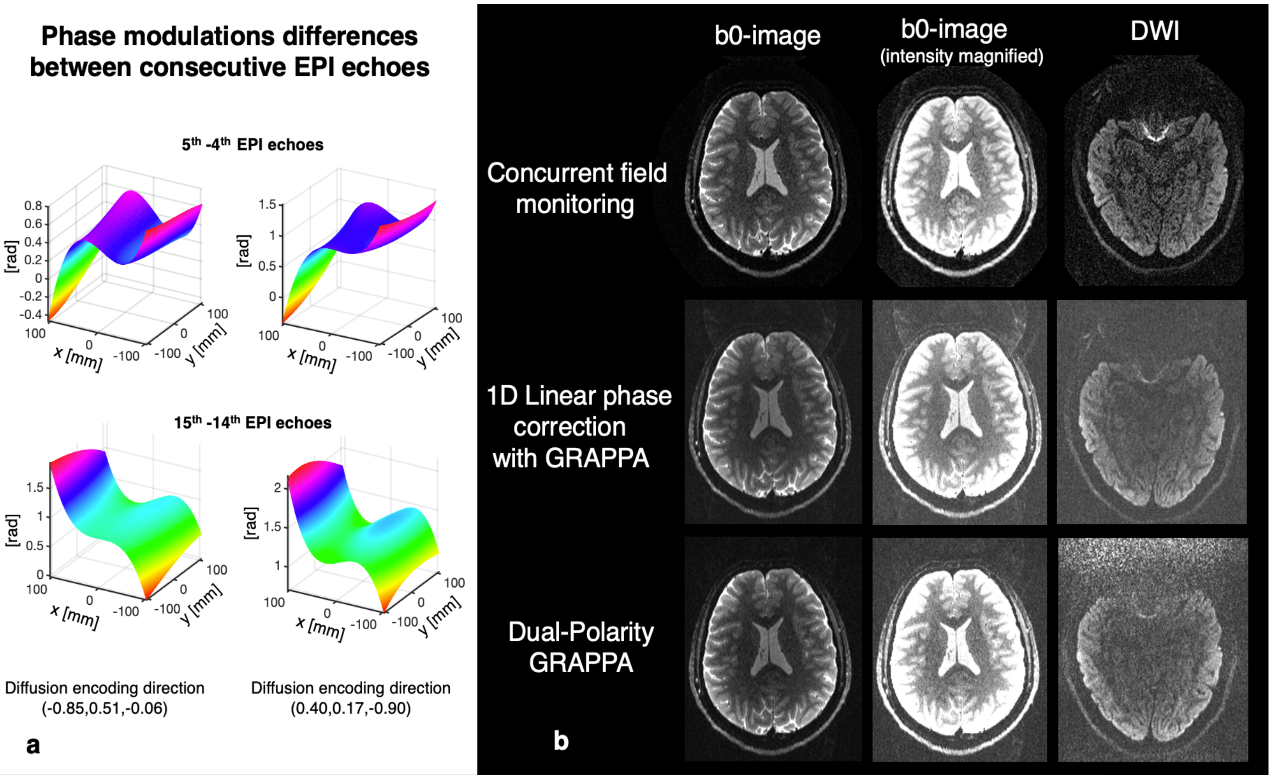

Supplementary Fig. S8 | Non-linear Nyquist ghosting removal. a, Spatial distribution of phase differences between consecutive EPI echoes at different time points of the image readout for different diffusion encoding directions. **b,** Nyquist ghosting removal results with concurrent field monitoring-based image reconstruction, one-dimensional linear phase correction with GRAPPA, and dual-polarity GRAPPA.

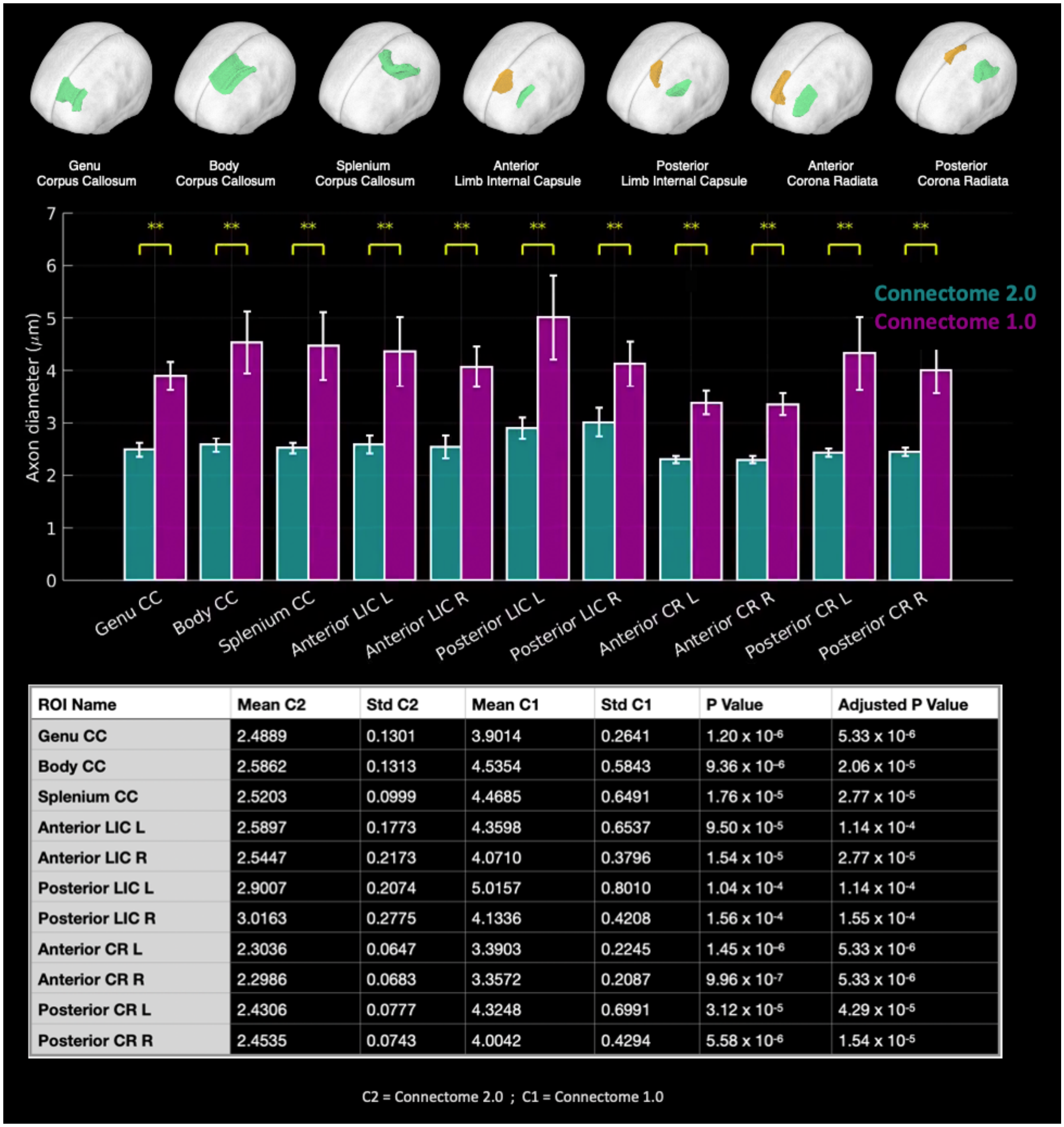

ROI Name	Mean C2	Std C2	Mean C1	Std C1	P Value	Adjusted P Value
Genu CC	2.4889	0.1301	3.9014	0.2641	1.20×10^{-6}	5.33×10^{-6}
Body CC	2.5862	0.1313	4.5354	0.5843	9.36×10^{-6}	2.06×10^{-5}
Splenium CC	2.5203	0.0999	4.4685	0.6491	1.76×10^{-5}	2.77×10^{-5}
Anterior LIC L	2.5897	0.1773	4.3598	0.6537	9.50×10^{-5}	1.14×10^{-4}
Anterior LIC R	2.5447	0.2173	4.0710	0.3796	1.54×10^{-5}	2.77×10^{-5}
Posterior LIC L	2.9007	0.2074	5.0157	0.8010	1.04×10^{-4}	1.14×10^{-4}
Posterior LIC R	3.0163	0.2775	4.1336	0.4208	1.56×10^{-4}	1.55×10^{-4}
Anterior CR L	2.3036	0.0647	3.3903	0.2245	1.45×10^{-6}	5.33×10^{-6}
Anterior CR R	2.2986	0.0683	3.3572	0.2087	9.96×10^{-7}	5.33×10^{-6}
Posterior CR L	2.4306	0.0777	4.3248	0.6991	3.12×10^{-5}	4.29×10^{-5}
Posterior CR R	2.4535	0.0743	4.0042	0.4294	5.58×10^{-6}	1.54×10^{-5}

C2 = Connectome 2.0 ; C1 = Connectome 1.0

Supplementary Fig. S9 | Mean and standard deviation of mean axon diameter index across representative white matter regions of interest. Bar plots of mean axon diameter index obtained from fitting the AxCaliber-SMT model in 11 white matter regions of interest averaged across 10 healthy adult subjects scanned on Connectome 2.0 (teal bars) and Connectome 1.0 (purple bars), respectively, corresponding to the data shown in Figure 7. The axon diameter index was estimated by fitting the AxCaliber-SMT model to the diffusion signal in each voxel. Asterisks indicate the statistically significant difference of axon diameter estimates between Connectome 2.0 and Connectome 1.0 ($p<0.001$, FDR-corrected). Regions of interest are displayed in the top row and include: the genu, body, and splenium of the corpus callosum (CC); the anterior and posterior limbs of internal capsule (LIC), and the anterior and posterior corona radiata (CR). L/R = left/right.

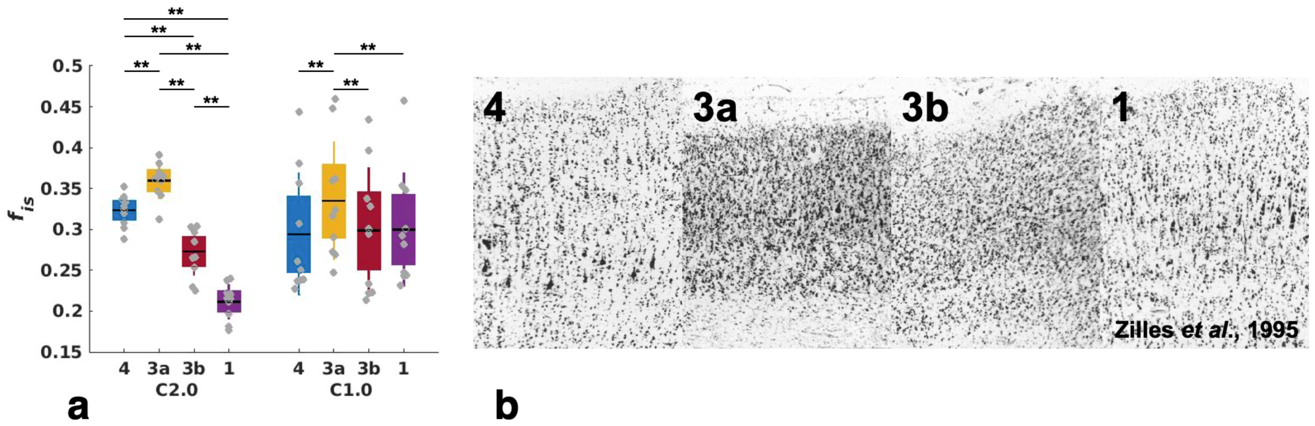

Supplementary Fig. S10 | Intra-soma signal fraction in subregions of sensorimotor cortex. a, Boxplots of intra-soma signal fraction (f_{is}) in regions of interest corresponding to Brodmann areas 4, 3a, 3b, and 1. The intra-soma signal fraction was estimated by fitting the SANDI model to diffusion signals in 10 healthy adult subjects scanned on Connectome 2.0 and Connectome 1.0 scanners. Boxplots show the mean (solid black line) and standard deviation (vertical line) averaged over all subjects for each scanner within region of interest, with individual datapoints represented by gray dots. * denotes statistically significant differences between region of interest averages where p-value < 0.05, and ** denotes statistically significant differences between region of interest averages where p-value < 0.005. All statistical comparisons were performed using the paired-samples t-test correcting for multiple comparisons using the FDR. **b**, Representative histological images from Nissl staining of Brodmann areas 4, 3a, 3b, and 1, demonstrating similar trends in cellular density as shown on the boxplots [7]. Supplementary Fig. S10b is adapted from [7] with permission from Blackwell Publishing LTD.

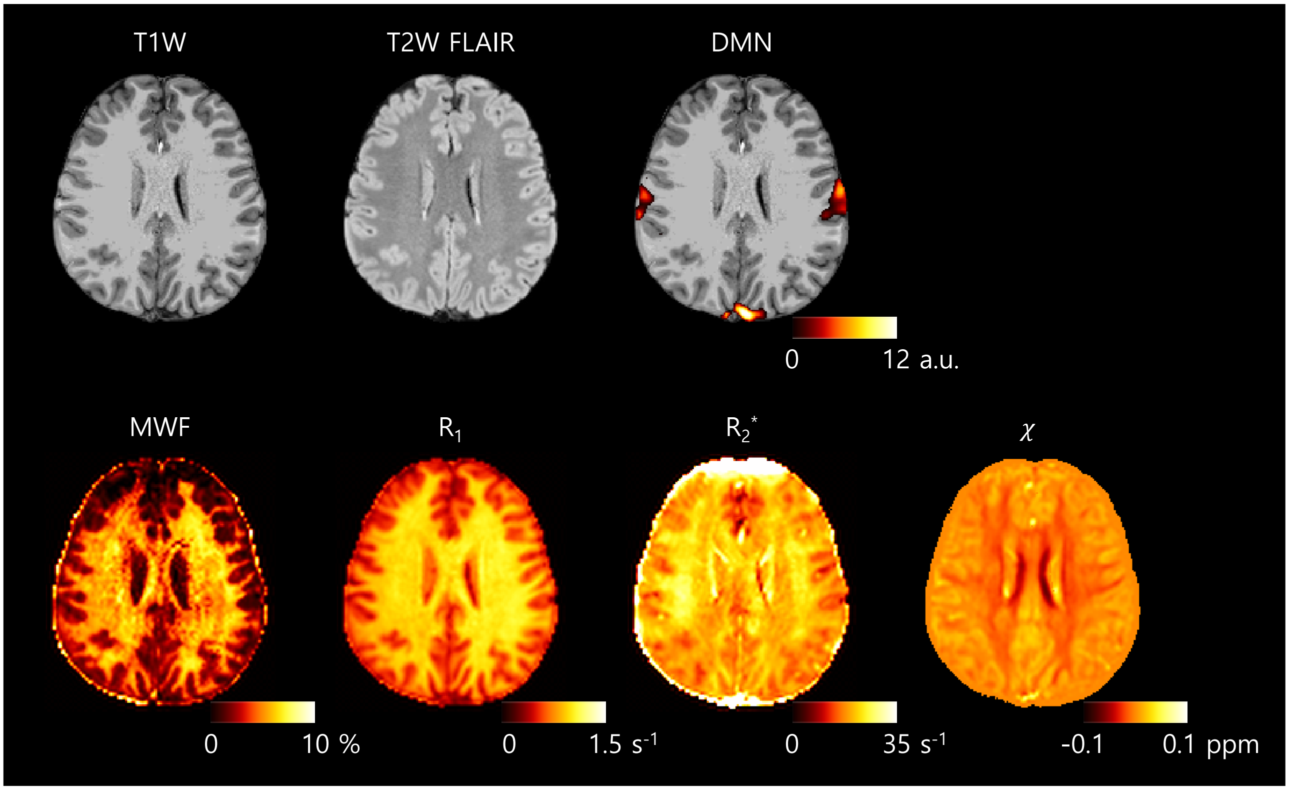

Supplementary Fig S11 l The multiparametric MRI obtained using the Connectome 2.0 scanner. The figure displays the T1-weighted image, T2-weighted fluid-attenuated inversion recovery (FLAIR) image, default mode network (DMN) map from resting-state functional MRI, myelin water fraction map, R1 map, R2* map, and quantitative susceptibility map (χ). See Supplementary Note S1 for acquisition details.

Supplementary Tables

Supplementary Table S1 l Summary of experimental PNS threshold characteristics measured for the Connectome 2.0 head gradient. We report both 0-to-peak (G_{stim}) and peak-to-peak minimum gradient amplitudes (ΔG_{min}) as well as chronaxie (t_{ch}) and SR_{min} (slope of the PNS threshold curve).

Gradient direction	G_{stim} (0-to-peak) [mT/m]	ΔG_{min} (peak-to-peak) [mT/m]	t_{ch} [us]	SR_{min} [T/m/s]
X	90.54	181.08	617.9	293.04
Y	88.23	176.45	496.8	355.16
Z	78.96	157.92	605.5	260.81
X±Y	86.55	173.11	444.1	389.81
Y±Z	73.83	147.66	446.6	330.60
X±Y+Z	78.40	156.79	405.8	386.37

Supplementary Notes

Supplementary Note S1. Acquisition details of multiparametric MRI obtained with the Connectome 2.0 scanner

Data acquisition for T1-weighted images

T1-weighted images were acquired by using a 3D magnetization-prepared rapid acquisition with gradient echo (MPRAGE) sequence with the following parameters: TR/TE/TI = 2500/3.36/1100 ms, isotropic voxel size = 1 mm, flip angle = 8°, 256 sagittal sections, in-plane image matrix = 256 × 256, and in-plan acceleration factor = 2.

Data acquisition for T2-weighted FLAIR images

T2-weighted fluid-attenuated inversion recovery (FLAIR) images were acquired with the following parameters: TR/TE/TI = 5000/392/1800 ms, isotropic voxel size = 1 mm, 160 sagittal sections, in-plane image matrix = 224 × 224, and in-plane acceleration factor = 2.

Data acquisition and image processing for resting-state functional MRI

For resting-state functional MRI, multiband gradient-echo EPI sequence was utilized to acquire a total of 350 volumes with the following parameters: TR/TE = 1090/30 ms, flip angle= 60°, isotropic voxel size = 2 mm, 66 axial slices, in-plane image matrix = 110 × 110, in-plan acceleration factor = 2, and SMS factor = 4. To correct for distortions, a gradient-echo field map was acquired with the same shimming and acquisition matrix. Functional data preprocessing was performed with FSL software (version 6.0.3; FMRIB Software Library, *https://fsl.fmrib.ox.ac.uk/fsl/fslwiki*) [8]. The FEAT software package within FSL was used for data preprocessing, which included linear registration to T1-weighted images, motion correction, field map unwarping, slice section timing correction, brain extraction, spatial smoothing with 5-mm full width at half maximum, and temporal filtering with a high-pass filter of 100 seconds [9]. The default mode network (DMN) map was obtained from independent component analysis (ICA) decomposition.

Data acquisition and image processing for relaxometry and myelin water imaging

Relaxometry data was acquired by using a monopolar 3D GRE sequence with 15 echoes, $TR/TE_1:\Delta TE:TE_{15}$ = 45ms/1.62:2.71:39.6ms, flip angle = [5,10,20,30,40,50,70]°, isotropic voxel resolution = 1.8 mm, a 5-fold acceleration with CAIPI under-sampling (CAIPI z-shift of 2) and acquisition time of 2 min 12 sec per flip angle. Sagittal acquisition was used to ensure whole-brain coverage and equal saturation of the inflowing blood with respect to brain tissue and to avoid slice profile artefacts. B_1 map was acquired using a turbo-flash protocol [10] to correct B_1 field inhomogeneities on the variable-flip-angle GRE data fitting.

Image registration was performed across all flip angles of the GRE data to mitigate minor head movements using a rigid body transformation with linear interpolation. B1 map was subsequently registered to the GRE space using a rigid body transformation between the B1 magnitude image and the first echo of the magnitude GRE image. To be able to utilize the diffusion microstructure information in myelin water imaging (DIMWI) [11], a transformation matrix from dMRI space to GRE data was derived by registering a b0 image of dMRI to the first echo GRE data based on rigid body transformation, which was used to transform the dMRI-derived resulting maps from native space to GRE space.

Single-compartment T_1, R_2* and quantitative susceptibility mapping

Both R_2* mapping and quantitative susceptibility mapping (QSM) were performed using the SEPIA toolbox (v1.2.2.6) [12] and only the flip angle of 20° (Ernst angle) data was used. R_2* mapping was performed using a closed-form solution on the magnitude multi-echo data [13], whereas both magnitude and phase data were used on the QSM reconstruction and the processing pipeline comprising (1) total field computation with ROMEO [14], (2) background field removal with VSHARP [15], and dipole field inversion using LP-

CNN [16]. Single-compartment T_1 maps were derived using the first echo of the GRE data with all flip angles based on DESPOT1 [17].

<u>Multi-compartment relaxometry for diffusion-informed myelin water imaging (MCR-DIMWI)</u>
A ball-and-stick model [18] was used to compute a maximum of 3 fiber directions per voxel and the intra-neurite volume fraction was estimated from SMT model [19] using only the diffusion time of 13 ms dMRI data. Voxel-wise fitting was performed with the MCR-DIMWI signal model using the dMRI-derived metrics and the variable-flip-angle, multi-echo GRE data to obtain the myelin water fraction (MWF) map [20]. A quadratic signal weighting strategy was used in the model fitting to mitigate the physiological noise that was more pronounced at later echoes [21].

Supplementary References

[1] Im K, Lee JM, Lyttelton O, Kim SH, Evans AC, Kim SI. Brain Size and Cortical Structure in the Adult Human Brain. Cerebral Cortex. 2008;18(9):2181-2191. doi:10.1093/CERCOR/BHM244

[2] Lee HH, Sodickson DK, Lattanzi R, Correspondence R, Lattanzi I. An analytic expression for the ultimate intrinsic SNR in a uniform sphere. Magn Reson Med. 2018;80(5):2256-2266. doi:10.1002/MRM.27207

[3] Foo TKF, Tan ET, Vermilyea ME, et al. Highly efficient head-only magnetic field insert gradient coil for achieving simultaneous high gradient amplitude and slew rate at 3.0T (MAGNUS) for brain microstructure imaging. Magn Reson Med. 2020;83(6):2356-2369. doi:10.1002/MRM.28087

[4] Lee SK, Mathieu JB, Graziani D, et al. Peripheral nerve stimulation characteristics of an asymmetric head-only gradient coil compatible with a high-channel-count receiver array. Magn Reson Med. 2016;76(6):1939-1950. doi:10.1002/MRM.26044

[5] Havel WJ, Nyenhuis JA, Bourland JD, et al. Comparison of rectangular and damped sinusoidal db/dt waveforms in magnetic stimulation. IEEE Trans Magn. 1997;33(5 PART 2):4269-4271. doi:10.1109/20.619732

[6] Den Boer J, Bakker R, Ham C, Smink J. Generalization to com-plex stimulus shape of the nerve stimulation threshold basedon existing knowledge of its relation to stimulus duration for rectangular stimuli. In: Proc. Intl. Soc. Mag. Reson. Med. ; 1999:108.

[7] Zilles' K, Schlaug2 G, Matelli3 M, et al. Mapping of human and macaque sensorimotor areas by integrating architectonic, transmitter receptor, MRI and PET data. *J Anat*. 1995;187(Pt 3):515. Accessed January 9, 2024. /pmc/articles/PMC1167457/?report=abstract

[8] Smith SM, Jenkinson M, Woolrich MW, et al. Advances in functional and structural MR image analysis and implementation as FSL. Neuroimage 2004;23(Suppl 1):S208–S219.

[9] Woolrich MW, Ripley BD, Brady M, Smith SM. Temporal autocorrelation in univariate linear modeling of FMRI data. Neuroimage 2001;14(6):1370–1386.

[10] Chung, S., Kim, D., Breton, E., Axel, L., 2010. Rapid B1+ mapping using a preconditioning RF pulse with TurboFLASH readout. Magnet Reson Med 64, 439–46. https://doi.org/10.1002/mrm.22423

[11] Chan, K.-S., Marques, J.P., 2020. Multi-compartment relaxometry and diffusion informed myelin water imaging – Promises and challenges of new gradient echo myelin water imaging methods. Neuroimage 221, 117159. https://doi.org/10.1016/j.neuroimage.2020.117159

[12] Chan, K.-S. & Marques, J. P. SEPIA—Susceptibility mapping pipeline tool for phase images. Neuroimage 227, 117611 (2021).

[13] Gil, R. et al. An in vivo study of the orientation-dependent and independent components of transverse relaxation rates in white matter. NMR in biomedicine 29, 1780–1790 (2016).

[14] Dymerska, B. et al. Phase unwrapping with a rapid opensource minimum spanning tree algorithm (ROMEO). Magn. Reson. Med. 85, 2294–2308 (2021).

[15] Li, W., Wu, B. & Liu, C. Quantitative susceptibility mapping of human brain reflects spatial variation in tissue composition. Neuroimage 55, 1645–1656 (2011).

[16] Lai, K.-W., Aggarwal, M., Zijl, P. van, Li, X., Sulam, J., 2020. Medical Image Computing and Computer Assisted Intervention – MICCAI 2020, 23rd International Conference, Lima, Peru, October 4–8, 2020, Proceedings, Part II. Lect Notes Comput Sc 12262, 125–135. https://doi.org/10.1007/978-3-030-59713-9_13

[17] Deoni, S. C. L., Rutt, B. K., Arun, T., Pierpaoli, C. & Jones, D. K. Gleaning multicomponent T1 and T2 information from steady-state imaging data. Magnetic resonance in medicine 60, 1372–1387 (2008).

[18] Hernandez, M., Guerrero, G.D., Cecilia, J.M., García, J.M., Inuggi, A., Jbabdi, S., Behrens, T.E.J., Sotiropoulos, S.N., 2013. Accelerating fibre orientation estimation from diffusion weighted magnetic resonance imaging using GPUs. PLOS ONE 8, e61892. https://doi.org/10.1371/journal.pone.0061892

[19] Kaden, E., Kelm, N.D., Carson, R.P., Does, M.D., Alexander, D.C., 2016. Multi-compartment microscopic diffusion imaging. Neuroimage 139, 346–359. https://doi.org/10.1016/j.neuroimage.2016.06.002

[20] Chan, K.-S., Marques, J.P., 2020. Multi-compartment relaxometry and diffusion informed myelin water imaging – Promises and challenges of new gradient echo myelin water imaging methods. Neuroimage 221, 117159. https://doi.org/10.1016/j.neuroimage.2020.117159

[21] Chan, K.-S., Chamberland, M., Marques, J.P., 2023. On the performance of multi-compartment relaxometry for myelin water imaging (MCR-MWI) – test-retest repeatability and inter-protocol reproducibility. Neuroimage 266, 119824. https://doi.org/10.1016/j.neuroimage.2022.119824
