# Supplementary material for: Ultra-high gradient connectomics and microstructure MRI scanner for imaging of human brain circuits across scales
Source: Nat Biomed Eng. 2025 Jul 16;10(2):309–24. doi: 10.1038/s41551-025-01457-x (PMC12920100; doi:10.1038/s41551-025-01457-x)
Supplement: Supplementary file 2 — Reporting Summary [file 41551_2025_1457_MOESM2_ESM.pdf]

nature portfolio

Corresponding author(s): Susie Yi Huang; Gabriel Ramos Llorden

Last updated by author(s): May 6, 2025

Reporting Summary

Nature Portfolio wishes to improve the reproducibility of the work that we publish. This form provides structure for consistency and transparency in reporting. For further information on Nature Portfolio policies, see our Editorial Policies and the Editorial Policy Checklist.

Statistics

For all statistical analyses, confirm that the following items are present in the figure legend, table legend, main text, or Methods section.

n/a	Confirmed	
☐	☒	The exact sample size (n) for each experimental group/condition, given as a discrete number and unit of measurement
☐	☒	A statement on whether measurements were taken from distinct samples or whether the same sample was measured repeatedly
☐	☒	The statistical test(s) used AND whether they are one- or two-sided<br>*Only common tests should be described solely by name; describe more complex techniques in the Methods section.*
☐	☒	A description of all covariates tested
☐	☒	A description of any assumptions or corrections, such as tests of normality and adjustment for multiple comparisons
☐	☒	A full description of the statistical parameters including central tendency (e.g. means) or other basic estimates (e.g. regression coefficient) AND variation (e.g. standard deviation) or associated estimates of uncertainty (e.g. confidence intervals)
☐	☒	For null hypothesis testing, the test statistic (e.g. F, t, r) with confidence intervals, effect sizes, degrees of freedom and P value noted<br>*Give P values as exact values whenever suitable.*
☒	☐	For Bayesian analysis, information on the choice of priors and Markov chain Monte Carlo settings
☒	☐	For hierarchical and complex designs, identification of the appropriate level for tests and full reporting of outcomes
☒	☐	Estimates of effect sizes (e.g. Cohen's d, Pearson's r), indicating how they were calculated

Our web collection on statistics for biologists contains articles on many of the points above.

Software and code

Policy information about availability of computer code

Data collection	All MRI data was acquired on Siemens Numaris X software version XA61A.
Data analysis	The following software packages were used for data analysis: FSL v.5.0.7, v.6.0.1, v.6.0.7.3; MRtrix3 v.3.0.3; FreeSurfer v.7.4.0; MATLAB 2023a.<br><br>Code for Theoretical Prediction of Shortest Echo Time (TE) is publicly available at GitHub: https://github.com/Connectome20/protocol_design_PGSE<br><br>Lee HH. Connectome20/protocol_design_PGSE: v1.0.0 (v1.0.0). Zenodo. https://doi.org/10.5281/zenodo.15312803. Published online 2025.<br><br>AxCaliber-SMT code is publicly available at GitHub: https://github.com/Connectome20/Biophysical-modeling/tree/main<br><br>Lee H, Ma Y. Connectome20/Biophysical-modeling: v.1.0.2 (v1.0.2). Zenodo. https://doi.org/10.5281/zenodo.15319985. Published online 2025.<br><br>Other image reconstruction, processing, and analysis code is available from the corresponding authors upon reasonable request.

For manuscripts utilizing custom algorithms or software that are central to the research but not yet described in published literature, software must be made available to editors and reviewers. We strongly encourage code deposition in a community repository (e.g. GitHub). See the Nature Portfolio guidelines for submitting code & software for further information.

Data

Policy information about availability of data

All manuscripts must include a data availability statement. This statement should provide the following information, where applicable:

- Accession codes, unique identifiers, or web links for publicly available datasets
- A description of any restrictions on data availability
- For clinical datasets or third party data, please ensure that the statement adheres to our policy

Raw and preprocessed diffusion-weighted images used to estimate axon diameter are publicly available at OpenNeuro: https://openneuro.org/datasets/ds006181/versions/1.0.0. All other data are available from the corresponding authors upon reasonable request

Research involving human participants, their data, or biological material

Policy information about studies with human participants or human data. See also policy information about sex, gender (identity/presentation), and sexual orientation and race, ethnicity and racism.

Reporting on sex and gender	Human adult volunteers of both sexes were recruited for the study. Sex was not considered relevant in the design of this technology development and validation study. Consent was not obtained for sharing of individual level data; therefore, only results at the group level are reported.
Reporting on race, ethnicity, or other socially relevant groupings	Race, ethnicity and other socially relevant groupings were not used in the manuscript as these variables were not considered applicable to this technology development and validation study.
Population characteristics	Healthy adult volunteers ages 23 to 45 years including both males and females were recruited.
Recruitment	The participants were recruited by investigators at Massachusetts General Hospital. There were no self-selection biases or other biases.
Ethics oversight	The study was approved by the Massachusetts General Brigham Institutional Review Board (IRB), and written informed consent was obtained prior to the examination.

Note that full information on the approval of the study protocol must also be provided in the manuscript.

Field-specific reporting

Please select the one below that is the best fit for your research. If you are not sure, read the appropriate sections before making your selection.

☒ Life sciences ☐ Behavioural & social sciences ☐ Ecological, evolutionary & environmental sciences

For a reference copy of the document with all sections, see nature.com/documents/nr-reporting-summary-flat.pdf

Life sciences study design

All studies must disclose on these points even when the disclosure is negative.

Sample size	Following phantom studies (not shown), 10 human subjects were scanned on the Connectome 2.0 scanner to demonstrate the imaging capabilities of the scanner. For comparison, data from 10 human subjects scanned on Connectome 1.0 were also included. Power analysis was not performed as this is a demonstration representative data on the Connectome 2.0 scanner and not a test of specific hypotheses. Sufficient subjects were recruited to demonstrate the benefits of the Connectome 2.0 scanner across a number of different analyses.
Data exclusions	No human subject data was excluded.
Replication	The results in Figure 5c and 5d were replicated in one other healthy adult volunteer (not shown). The experiments in Figure 6 were performed independently in 10 healthy adult participants and replicated in another 10 healthy adult volunteers
Randomization	Randomization was not applicable, as the goal of the study was imaging validation using the novel MRI scanner and there were no groups in this study.
Blinding	Blinding was not relevant to the study, as the goal was imaging validation using the Connectome 2.0 MRI scanner. Human subject data were anonymized per HIPAA rules.

Reporting for specific materials, systems and methods

We require information from authors about some types of materials, experimental systems and methods used in many studies. Here, indicate whether each material, system or method listed is relevant to your study. If you are not sure if a list item applies to your research, read the appropriate section before selecting a response.

Materials & experimental systems

n/a	Involved in the study	
☒	☐	Antibodies
☒	☐	Eukaryotic cell lines
☒	☐	Palaeontology and archaeology
☒	☐	Animals and other organisms
☒	☐	Clinical data
☒	☐	Dual use research of concern
☒	☐	Plants

Methods

n/a	Involved in the study	
☒	☐	ChIP-seq
☒	☐	Flow cytometry
☐	☒	MRI-based neuroimaging

Magnetic resonance imaging

Experimental design

Design type: Diffusion MRI

Design specifications: T1- and diffusion-weighted were acquired in human subjects. Resting-state fMRI were performed in human subjects. No performance/task-based fMRI or clinical trials were performed.

Behavioral performance measures: No behavioral tasks were performed.

Acquisition

Imaging type(s): Diffusion imaging

Field strength: 3T

Sequence & imaging parameters: All diffusion-weighed images were acquired with a monopolar 2D PGSE-EPI sequence. Specific acquisitions details for each experiments are reported in Methods Section.

Area of acquisition: Whole brain

Diffusion MRI ☒ Used ☐ Not used

Parameters: Specific details for each diffusion experiment are provided in Method Section.

Preprocessing

Preprocessing software: Processing software includes FSL and FEAT (FMRIB Sofware Library), MRtrix3, and custom code written in Matlab (version 2023a)

Normalization: Maps of tissue microstructure metrics were transformed from each individual's native diffusion space to the common MNI152 space. Specific details can be found at Method section.

Normalization template: MNI152 space

Noise and artifact removal: Tools 'top-up' and 'eddy' from FSL were used for susceptibility and eddy-current induced distortion correction. Matlab custom code were used to correct for gradient non-linearity correction. Specific details can be found at Method section.

Volume censoring: Not applicable.

Statistical modeling & inference

Model type and settings: Not applicable.

Effect(s) tested: Not applicable.

Specify type of analysis: ☐ Whole brain ☐ ROI-based ☒ Both

Anatomical location(s): White matter ROIs were obtained from the Jon Hopkins University (JHU) white matter atlas. Cortical regions were obtained from FreeSurfer automated labeling.

Statistic type for inference<br>(See Eklund et al. 2016)	Statistical hypothesis testing with two-sided t-tests.
Correction	False discovery rate (Benjamini-Hochberg correction)

Models & analysis

n/a	Involved in the study
☒	☐ Functional and/or effective connectivity
☒	☐ Graph analysis
☒	☐ Multivariate modeling or predictive analysis
